# Machine learning applied to global scale species distribution models

**DOI:** 10.1038/s41598-025-20797-x

**Published:** 2025-10-27

**Authors:** Alba Fuster-Alonso, Jorge Mestre-Tomás, Jose Carlos Baez, Maria Grazia Pennino, Xavier Barber, Jose María Bellido, David Conesa, Antonio López-Quílez, Jeroen Steenbeek, Villy Christensen, Marta Coll

**Affiliations:** 1 Present Address: Renewable Marine Resources Department, Institute of Marine Sciences (ICM)-CSIC, Barcelona, 08003 Spain; 2 Department of Statistics and Operations Research (VaBar), Universitat de València, Valencia, Spain; 3 Department of Applied Statistics and Operational Research, Universitat Politècnica de València (UPV), 46022 Valencia, Spain; 4 Spanish Institute of Oceanography (IEO)-CSIC, Oceanographic Center of Málaga, Fuengirola, 29640 Spain; 5 Ibero-American Institute for Sustainable Development (IIDS), Autonomous University of Chile, Av. Alemania 1090, Temuco, 4810101 Araucanía Region Chile; 6 Spanish Institute of Oceanography (IEO) - CSIC, Oceanographic Center of Madrid, C. Del Corazón de María, 8, 28002 Madrid, Spain; 7 Center of Operations Research, Miguel Hernández University (UMH), Elche, Spain; 8 Spanish Institute of Oceanography (IEO)-CSIC, Oceanographic Center of Murcia, San Pedro del Pinatar, Murcia, Spain; 9 Ecopath International Initiative (EII), Barcelona, Spain; 10 Institute for the Oceans and Fisheries, University of British Columbia, Vancouver, Canada

**Keywords:** Marine turtles, Global scale, Long-term prediction, Spatial distributions, Environmental change, Machine learning, BART, Simulation, Ecology, Ecological modelling

## Abstract

Species Distribution Models (SDMs) are widely used in ecology to analyze historical and future patterns of marine species distributions. Given the growing impact of climate change, predicting potential shifts in species ranges has become a key challenge. In this study, we apply Bayesian Additive Regression Trees (BART), a non-parametric machine learning algorithm, to estimate and forecast the global distribution of marine turtle species under different climate change scenarios. We model both individual species and their combined functional group, assess their historical and future habitat suitability, and examine the contribution of key environmental predictors. To evaluate BART’s performance, we conduct a simulation study under two contrasting distributional scenarios: a cosmopolitan and a persistent species. We also test the sensitivity of BART to pseudo-absence data and compare its performance with MaxEnt and Generalized Additive Models (GAMs). Results indicate that BART performs slightly better overall, particularly under pseudo-absence settings, showing higher accuracy and more stable sensitivity and specificity. These findings highlight BART as a reliable alternative for long-term, global-scale species distribution modeling in marine systems.

## Introduction

The impact of climate change on marine ecosystems has been increasingly recognized as a global phenomenon, with numerous studies highlighting its effects worldwide^1–4^. As environmental conditions continue to change, marine species must adapt and potentially shift their distributions to areas with more suitable conditions for their survival and reproduction^1,5–11^. Therefore, understanding the present spatiotemporal distribution of marine species and accurately predicting their future changes is a critical challenge in the current context of global warming^12,13^.

For this reason, macroecological approaches have gained importance in recent decades^14–21^, providing broad insights into large-scale patterns of species distributions^22^. These global approaches are essential for evaluating climate change^12,13,23^, contributing to the development of effective management strategies with global policy objectives^14,24^.

Species Distribution Models (SDMs), also referred to as Ecological Niche Models (ENMs) or Habitat Suitability Models (HSMs), are widely used tools for understanding species and community distributions in space and their potential shifts over time^25,26^. The terminology often depends on the focus of the study: SDMs emphasize spatial distributions, while ENMs highlight the ecological and niche drivers underlying those distributions^27,28^. In this study, we use SDMs as a general term, encompassing all approaches that estimate species’ ecological requirements to predict their distributions across space and time. However, we acknowledged the difference between ENMs and SDMs^27,28^. While ENMs aim to estimate a species’ fundamental ecological niche based on its physiological limits and ecological tolerances, our focus is on SDMs that predict potential habitat suitability under changing environmental conditions, particularly climate change scenarios.

Therefore, SDMs allow researchers to link information about the presence/absence of species to key environmental drivers to predict where and how a species is likely to be present in unsampled areas or time periods^29,30^. SDMs have provided insights into species distributions’ patterns, species-environment relationships, and potential habitat suitability^31–35^.

Since SDMs have been used in the context of range shifts for the past 30 years, numerous approaches and techniques have been developed, each with unique strengths and limitations. Recently, there has been a notable trend towards using machine learning methods within SDMs due to their strong predictive capacity, flexibility in handling complex and non-linear relationships, and ability to incorporate large dataset^30,36^.

A promising and innovative alternative to traditional SDMs regression tree methods is the Bayesian Additive Regression Trees (BART) approach^37,38^. BART is a non-parametric Bayesian regression approach that is based on a sum-of-trees model and is fundamentally an additive model with multivariate components^38^. This methodology offers some advantages over conventional SDMs, making it an alternative choice for ecological research.

One of the key advantages of BART over traditional classification tree methods is its incorporation of prior distributions, which limits the influence of individual trees on the overall model. By reducing dependence among trees, BART mitigates the issue of overfitting commonly associated with regression trees^39^. This ensures that no single tree dominates the predictions, allowing the model to strike a balance between constructing accurate trees and maintaining the flexibility needed to predict species distributions in unsampled areas or future time periods. As a result, this feature enhances the robustness and reliability of the model’s predictions^40^. Furthermore, the Bayesian framework of the BART method enables the estimation of prediction uncertainty, a feature that is generally absent or computationally expensive in most traditional species distribution modeling techniques^41^.

In general, BART has been used in the context of SDMs, but existing studies have been limited to local or regional scales^41–48^. To date, there has been no comprehensive application of BART to a broader and larger spatial extent for estimating the effects of climate change on marine species. Therefore, we present BART as an alternative for modeling on a global scale that allows the user to update data or include different drivers and to have a deep understanding of uncertainty. There is a significant need for more research that directly addresses global modelling approaches, highlighting the novelty and relevance of our contribution to the field. For this reason, our main goal is to apply BART on a global scale for the estimation and prediction of spatial-temporal distributions of different marine species and their relationship with environmental variables.

Our hypothesis is that BART may be a powerful approach to predict historical and future scenarios about the distribution of target species and functional groups, as well as their relationship with key environmental variables, on a global scale. To test our hypothesis, we conducted a case study on the functional group of marine turtles to assess the applicability of BART. This group includes all seven existing species of marine turtles, which are distributed very differently in the marine environment^49,50^. Moreover, ongoing research discuss how marine turtles face imminent threats to their survival in the wake of climate change^51–53^. This information, combined with their different distribution patterns, makes marine turtles an ideal functional group for testing the effects of climate change on a global scale. The study we present here applies the BART method to obtain native ranges, potential habitat, relations with environmental variables, and distribution projections under different future scenarios of climate change using outputs from Earth System Models (ESM) freely available through the Inter-Sectoral Impact Model Intercomparison Project (ISIMIP), an initiative and framework to establish consistent climate impact simulations,^54,55^ and Fish-MIP initiative (https://fish-mip.github.io/). Due to the uncertainty related to the predictions of climate change effects, we considered two different ESM set of drivers for our case of study: GFDL-ESM4 and IPSL-CM6A-LR and two different climate scenarios based on carbon emission.

Although a real case study can shed light on the predictive capability of a model, the validation associated with such study is contingent upon errors in observations, as we lack knowledge of the true current or future distribution of the species. For this reason, a simulation protocol has also been developed, allowing us to investigate the results of a hypothetical species. Then, through various random samplings of simulations, we can obtain presence, absence and pseudo-absence data to fit the model and assess its predictive capacity^56^. In this study, we assess two different simulation scenarios: one considers a hypothetical species that is spread over the entire domain (‘cosmopolitan’ species), while the second scenario considers a species that remains permanently in a specific area (‘persistent’ species). To evaluate the performance and robustness of the BART model, we compare its results with those obtained using two widely applied species distribution modeling techniques: MaxEnt and Generalized Additive Models (GAMs).

## Results

### Overview of global BART analysis workflow

**Fig. 1:**
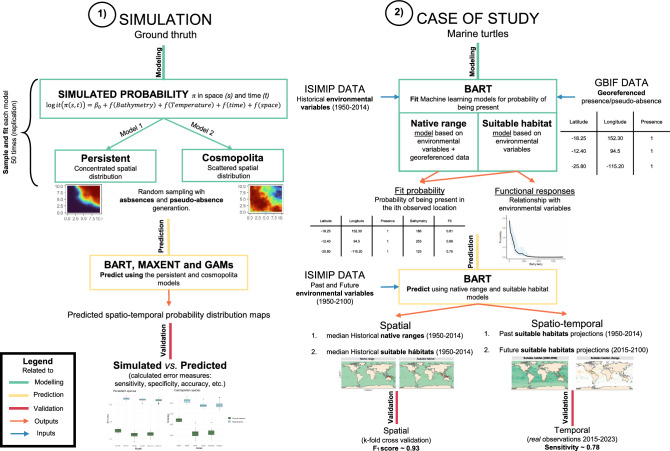
Overview of the methodological framework used in this study. The workflow is divided into two components: (1) a simulation study with two hypothetical species distribution patterns (cosmopolitan and persistent), where presence, absence, and pseudo-absence data were generated to evaluate and compare the performance of three modeling approaches (BART, MaxEnt, and GAMs) across 50 replicates; and (2) a real case study using georeferenced records of marine turtles, where the BART model was used to predict species distributions based on historical and future environmental conditions. Model performance was assessed using spatial and temporal validation metrics, including sensitivity, specificity, accuracy and $F_{1}$ score.

Our study is divided into two sections: (1) a simulation study, where we illustrate the performance of BART in a presence/absence and pseudo-absences simulation and modeling framework, comparing it to other models such as MaxEnt and GAMs; and (2) a case study, where we present the results obtained from BART for functional group of the marine turtles (Fig. 1).

Therefore, section 1) aims to assess the capacity of BART to project in space and time the distribution of different species. For this purpose, we simulate two different scenarios of probability according to the behavior of a species: first, a cosmopolitan species, where the species is dispersed over the entire domain, and second, a persistent species, where we observe a concentrated spatial distribution. Then, we perform 50 different random samplings to obtain presence/absence and pseudo-absences data to fit 50 different models and predict using BART, MaxEnt and GAMs. This allows us to obtain error measures of the predicted spatio-temporal distribution with respect to the simulated ground truth.

On the other hand, section 2) is based on applying BART on a global scale using the marine turtle’s functional group as a case study. Hence, we develop two different models: the native range model and the suitable habitat model. The main difference between these two models is that for the native range, we include the latitude and longitude of observations as covariates in the model, while the suitable habitat model is only based on environmental covariates. The inputs used are georeferenced occurrence data from GBIF and historical, past, and future projections of environmental variables from ISIMIP. The common output of the native range and suitable habitat models is a map representing the historical spatial distribution from 1950-2014 of each individual species. Then, using the suitable habitat model, we projected the distribution for each year from 1950 to 2100. The results are validated using a k-fold cross-validation and real observations (2015–2023).

### Simulation

Regarding the simulation framework, both simulation scenarios of the occurrence data of a hypothetical cosmopolitan and persistent species were developed by considering several effects: (1) a spatial-temporal effect, (2) a bathymetric effect, (3) a temperature effect, and (4) a temporal trend. Therefore, a series of parameters was set for all terms in Eqs. (1) and (2) (see the Methods section). Additionally, we assessed the performance of BART, MaxEnt and GAMs to the generation of pseudo-absences for both scenarios: (1) cosmopolitan, and (2) persistent species.

#### Cosmopolitan species

**Fig. 2:**
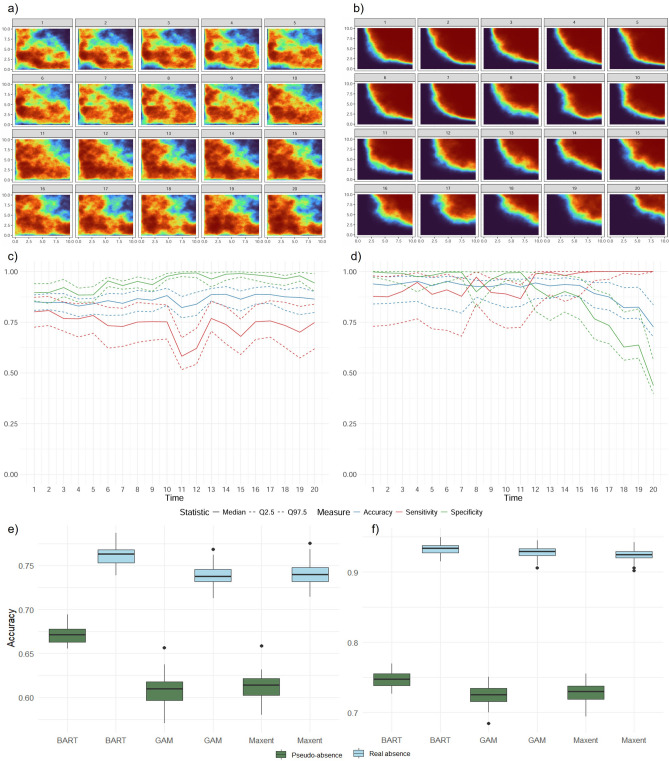
Figures (**a**) and (**b**) are the simulation of the probability distribution in space and time for a cosmopolitan and persistent species scenarios respectively. The time window is 20 years, and we can observe changes over space and time. Figures (**c**) and (**d**) are the results of BART models sensitivity, specificity, and accuracy for the cosmopolitan and persistent scenarios respectively. We have calculated the mean and quantiles (0.025 and 0.975) over the 50 replications conducted. Figures (**e**) and (**f**) are the results of accuracy for MaxEnt, GAMs, and BART models perfomance including the real absence and pseudo-absence scenarios.

The evolution of the simulated probability of presence for a cosmopolitan species is characterized by a broad and continuous distribution throughout space and time, over a 20-year period across a hypothetical landscape (Fig. 2a). The simulation incorporates a correlated spatial effect, with range $r = 3.5$, variance $\sigma = 1$, and temporal correlation $\rho = 0.7$; a second-degree polynomial for the bathymetry effect (constant over time), with coefficients $\beta _{1X_{1}(s)} = -1.5$ and $\beta _{2X_{1}(s)} = -1.1$; and bathymetry values ranging from 0 to 800 meters, calculated using the formula $100 \cdot \log (xy + 1)$, where *x* and *y* are the spatial coordinates; temperature was modeled as a linear effect using the formula $\sqrt{y+1} + 10$, and since temperature is a dynamic variable, we added 0.5 units per year to represent a temporal increase; the temporal trend was simulated using an autoregressive model of order 1 (AR1), with $\rho _{t} = 0.7$. All predictor terms (as described in Equation 1) were summed and transformed to the probability scale (ranging from 0 to 1) using the inverse of the logit link function.

After simulating the probabilities, we performed 50 samplings to obtain the presence/absence data for fitting the BART model (Eq. 3). Then we simulate from a Bernouilli distribution to distinguish between presence and absence according to the simulated probability. It should be noted that, for each sampling, we selected a total of 50 observations. Once we had the presence/absence data, we were able to fit the models and then make predictions across both spatial and temporal dimensions. In the repository, we can observe all the predictions in space and time for each replica. Note that we excluded data from years 18, 19, and 20 during the fitting process. This exclusion allowed for the subsequent projection of the entire distribution into the future.

Figure 2c presents the median, along with the 0.025 and 0.975 quantiles, of the validation measures (sensitivity, specificity, and accuracy) for all models fitted with BART. We observe high specificity at the beginning of the study period, while the sensitivity is lower, particularly in the 11-year period. This pattern remains constant for 20 years, where the specificity increases, leading to a decrease in sensitivity. However, when we assess accuracy, it consistently remains close to one, around 0.8, throughout the entire period. In Figure S13 of the supplementary material, we observe the values of sensitivity, specificity, and accuracy for the pseudo-absence scenario, where the results follow the same pattern as those in Fig. 2c for all metrics but with lower values, although accuracy always remains higher than 0.5, with values around 0.7. Figure 2e illustrates the accuracy of MaxEnt, GAMs, and BART models using real and pseudo-absence data, with BART achieving the highest accuracy in both scenarios. Specifically, BART reached an accuracy of 0.78 with real absences and 0.68 with pseudo-absences. MaxEnt showed an accuracy of 0.74 with real absences and 0.62 with pseudo-absences, while GAMs had the lowest performance, with values 0.73 and 0.61, respectively.

#### Persistent species

In contrast to the cosmopolitan scenario, the distribution of a persistent species is spatially restricted and temporally consistent, reflecting strong site fidelity and environmental specialization (Fig. 2b). This simulation was based on Eq. (2), with adjusted parameter values, where the range and variance of the spatio-temporal effect were set to 5.6 and 1, respectively; the autoregressive coefficient for the spatio-temporal component was $\rho _{U} = 0.1$, and for the temporal trend, $\rho _{t} = 0.7$; bathymetry and temperature covariates followed the same functional forms as in the cosmopolitan scenario, but with different fixed coefficients, where bathymetry was modeled with $\beta _{1X_{1}(s)} = 7.5$, indicating a strong positive association, and temperature with $\beta _{2X_{2}(s,t)} = -0.8$, reflecting a moderate negative effect. Based on these probability maps, we performed 50 replicate samplings of presence/absence and pseudo-absence data, following the same methodology as used for the cosmopolitan species.

Figure 2d displays the median, along with the 0.025 and 0.975 quantiles, of the validation metrics (sensitivity, specificity, and accuracy) across all replications for the BART model. In this scenario, all metrics remained consistently high throughout most of the time series, particularly in years where predictions were limited to unsampled locations but not extrapolated in time. However, a decline in sensitivity, specificity and accuracy was observed in the final years (18–20), which were used for temporal extrapolation. Despite this decline, accuracy remained above 0.75 even in the projection years. Supplementary Figure S14 shows the corresponding metrics under the pseudo-absence scenario. While the trends were similar to those in Fig. 2d, the values were slightly lower across all metrics. Figure 2f displays the accuracy of MaxEnt, GAMs, and BART models using both real and pseudo-absence data. Again, BART outperformed the other models in both scenarios. Specifically, BART achieved an accuracy of 0.94 with real absences and 0.75 with pseudo-absences. MaxEnt reached 0.91 and 0.73, respectively, while GAMs showed the lowest accuracy values, with 0.92 using real absences and 0.72 with pseudo-absences.

### Case study

#### Species predictions

Here, we present the results for the present period (1950–2014) and the predicted changes for the future (2015–2100) for two species, along with their response functions to environmental variables. The two species chosen are the Australian flatback sea turtle *Natator depressus* and the leatherback sea turtle *Dermochelys coriacea*, both with very different spatial distributions. The first species is distributed mainly along the Australian coast, while the second species is widely distributed throughout the world^49,50^. The detailed results and figures for the remaining species can be found in the supplementary material (see Section 2 species predictions).

**Fig. 3:**
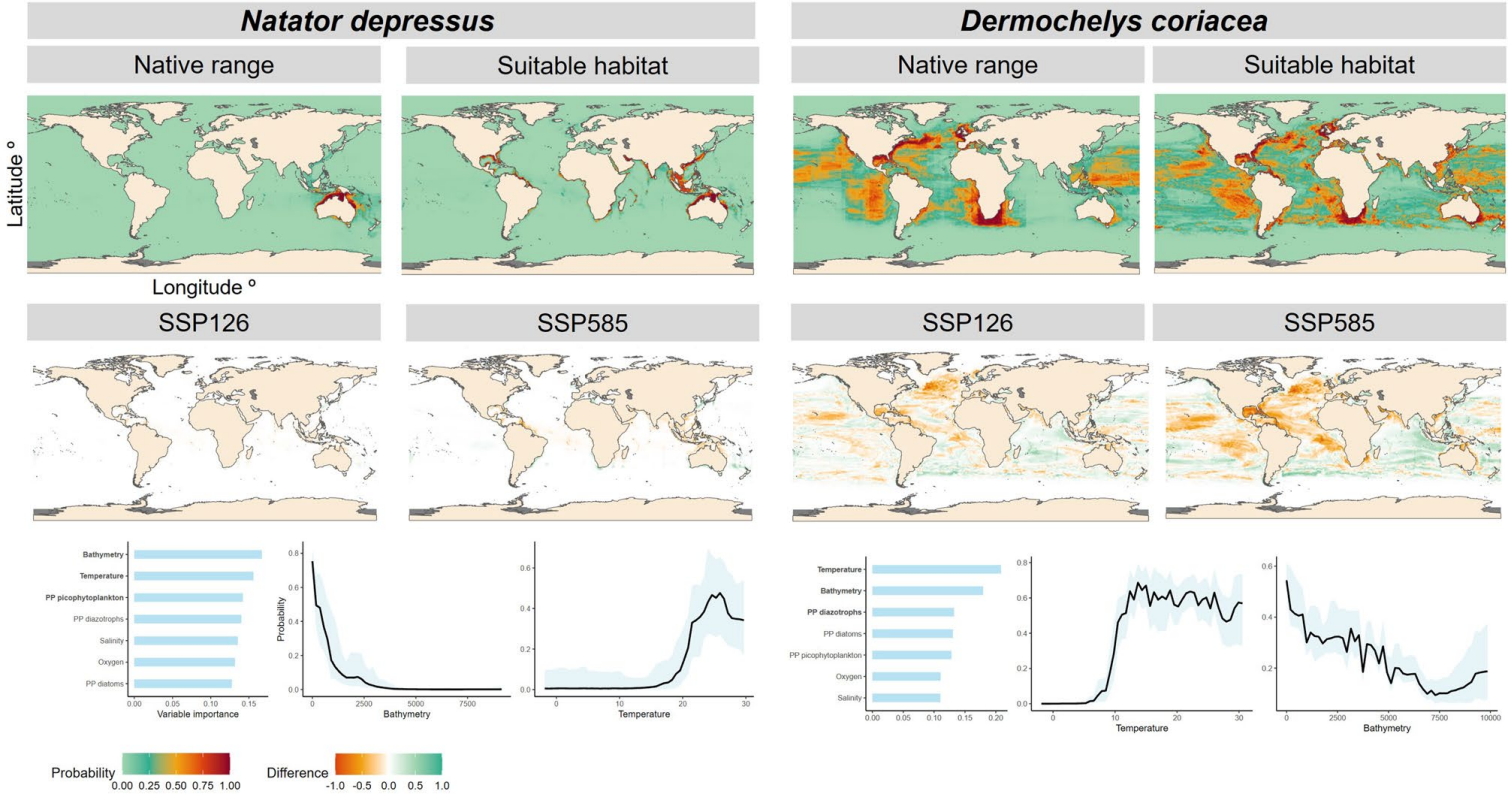
The first row illustrates the native range and mean probability of suitable habitat presence for *Natator depressus* and *Dermochelys coriacea*, while the second row shows the difference between historical and future suitable habitats under two climate change scenarios: SSP1-2.6 and SSP5-8.5. The third row represents the contribution of bathymetry, temperature, and other drivers to the model, as well as the functional responses. All maps correspond to results from the GFDL-ESM4 model.

Since our analysis follows a Bayesian approach, each pixel in the resulting maps contains a full posterior predictive distribution rather than a single point estimate. This allows for a more comprehensive representation of species distributions by incorporating uncertainty directly into the predictions. In the figures presented in the results section, we show the posterior mean probability of presence to summarize these distributions (Figs. 3 and 5). In section 2 of the supplementary material, we present both the posterior mean and the associated uncertainty, represented by the 2.5% and 97.5% quantiles.

Results are shown for both the native range and the suitable habitat models, producing different spatial distributions predictions (Fig. 3). Native ranges show much narrower predicted areas of high probability compared to suitable habitats, which is expected given that native ranges reflect historically observed distributions and are limited by the occurrence data used as covariate in the model calibration. Suitable habitat models, on the other hand, highlight regions with favorable environmental conditions, even beyond the areas where the species has been recorded. Furthermore, in section 2 of the supplementary material we illustrate the results of both models (native range and suitable habitat) for each of the ESMs (GFDL-ESM4 and IPSL-CM6A-LR ESM), resulting in very similar probability maps for both species historically.

The difference between the posterior mean probability of presence for the future period (2090–2099) and the historical baseline (1950–2014) is used to illustrate changes in suitable habitat under two climate scenarios, SSP126 and SSP585 (Fig. 3). These difference maps highlight areas of potential habitat gain or loss. The maps of predicted probabilities for the future period are provided in section 2 of the supplementary material.

For the species *Natator depressus*, our results project a reduction in potential habitat along the northern coast of Australia and gains in the Northern Hemisphere (Fig. 3). In the case of *Dermochelys coriacea*, a loss of potential habitat is observed in the Northern Hemisphere with GFDL-ESM4, while gains are projected in the south (Fig. 3). For both species, the IPSL-CM6A-LR ESM projects greater range expansions, particularly in northern regions, whereas the GFDL-ESM4 model indicates more pronounced losses in the Atlantic Ocean (see section 2 of the supplementary material). Moreover, under the SSP126 scenario, gains and losses appear less distinct compared to the SSP585 scenario for both species (Fig. 3).

Figure 3 also shows the contribution of environmental variables and the estimated non-linear relationships. For both species, the two variables that contribute the most to the model are bathymetry and sea surface temperature (Fig. 3). However, it is worth noting that all variables in the model have a similar contribution (Fig. 3). In the non-linear relationships with the response variable, it can be seen that for bathymetry, both species have their optima at low bathymetric values (Fig. 3). Whereas for SST, the behavior is sigmoidal, their distribution increases until reaching a maximum and then starts to decrease (Fig. 3).

In the same way, the native ranges and suitable habitats for other five species are presented in section 2 species predictions (Figures S4, S5 and S6) of the supplementary material. Notably, *Caretta caretta* appears to have a distribution concentrated in sub-tropical latitudes and along the west coast of Africa (Figure S2 in the supplementary material). *Lepidochelys olivacea* seems to have a more southern distribution compared to *Caretta caretta*, along with *Eretmochelys imbricata* and *Chelonia mydas* (Figure S2, S3 and S4 of the supplementary material). In contrast, *Lepidochelys kempii* exhibits a more confined distribution in the Atlantic Ocean and Europe.

Figures S6, S7, and S8 of the supplementary material have information on suitable habitat changes for these five species, as well as their contributions and relationships with environmental variables (Figures S9, S10, and S11 in the supplementary material). Most species appear to experience significant losses around tropical zones, except for *L. kempii*, where the losses are concentrated on the Atlantic North Coast. The spatial results obtained from both ESMs seem to agree in general terms, with the SSP585 climate scenario showing much more pronounced losses and gains for all species.

Global species richness hotspots for marine turtles are shown, with values ranging from 0 to 6 (Figure S12). These hotspots indicate regions where multiple species coexist, with warmer colors representing higher richness. The maximum observed value is 6, as it is biologically unfeasible for all seven species of marine turtles to overlap in the same area. This limitation is due to the restricted distributions of certain species, such as *N. depressus*, which is confined to the Indo-Pacific region, and *L. kempii*, which occurs only in the Atlantic Ocean.

Median values for four summary statistics were computed for each ESM and species (Table 1). Results show that across all metrics, the model performance is notably strong, with most values close 1. While there is a slight performance difference favoring GFDL-ESM4 ESM over IPSL-CM6A-LR, this discrepancy is small. Alternatively, to validate our future projections, we compared maps projected for the period 2015–2023 with actual observations obtained from GBIF during the same time frame. This comparison allowed us to calculate the sensitivity for each climate scenario and ESM. Our results indicate high levels of sensitivity, with GFDL-ESM4, reaching 0.77 and 0.79 for the SSP126 and SSP585 scenarios, respectively. IPSL-CM6A-LR model also demonstrates strong performance, achieving sensitivity values of 0.75 for both SSP126 and SSP585 climate scenarios.

**Table 1:** Different error measures for each species and ESM results (GFDL-ESM4 and IPSL-CM6A-LR). We have calculated sensitivity, specificity, accuracy, and $F_{1}$ score.

GFDL-ESM4				
Species	Sensitivity	Specificity	Accuracy	$F_{1}$ score
*Natator depressus*	0.98	0.98	0.98	0.98
*Dermochelys coriacea*	0.80	0.84	0.82	0.82
*Caretta caretta*	0.94	0.90	0.92	0.92
*Lepidochelys olivacea*	0.92	0.93	0.92	0.92
*Chelonia mydas*	0.93	0.92	0.92	0.92
*Lepidochelys kempii*	0.97	0.98	0.98	0.98
*Eretmochelys imbricata*	0.95	0.94	0.95	0.95

IPSL-CM6A-LR				
*Natator depressus*	0.97	0.97	0.97	0.97
*Dermochelys coriacea*	0.73	0.83	0.78	0.77
*Caretta caretta*	0.90	0.88	0.90	0.90
*Lepidochelys olivacea*	0.90	0.91	0.90	0.90
*Chelonia mydas*	0.91	0.89	0.90	0.90
*Lepidochelys kempii*	0.96	0.97	0.96	0.96
*Eretmochelys imbricata*	0.93	0.94	0.93	0.93

**Fig. 4:**
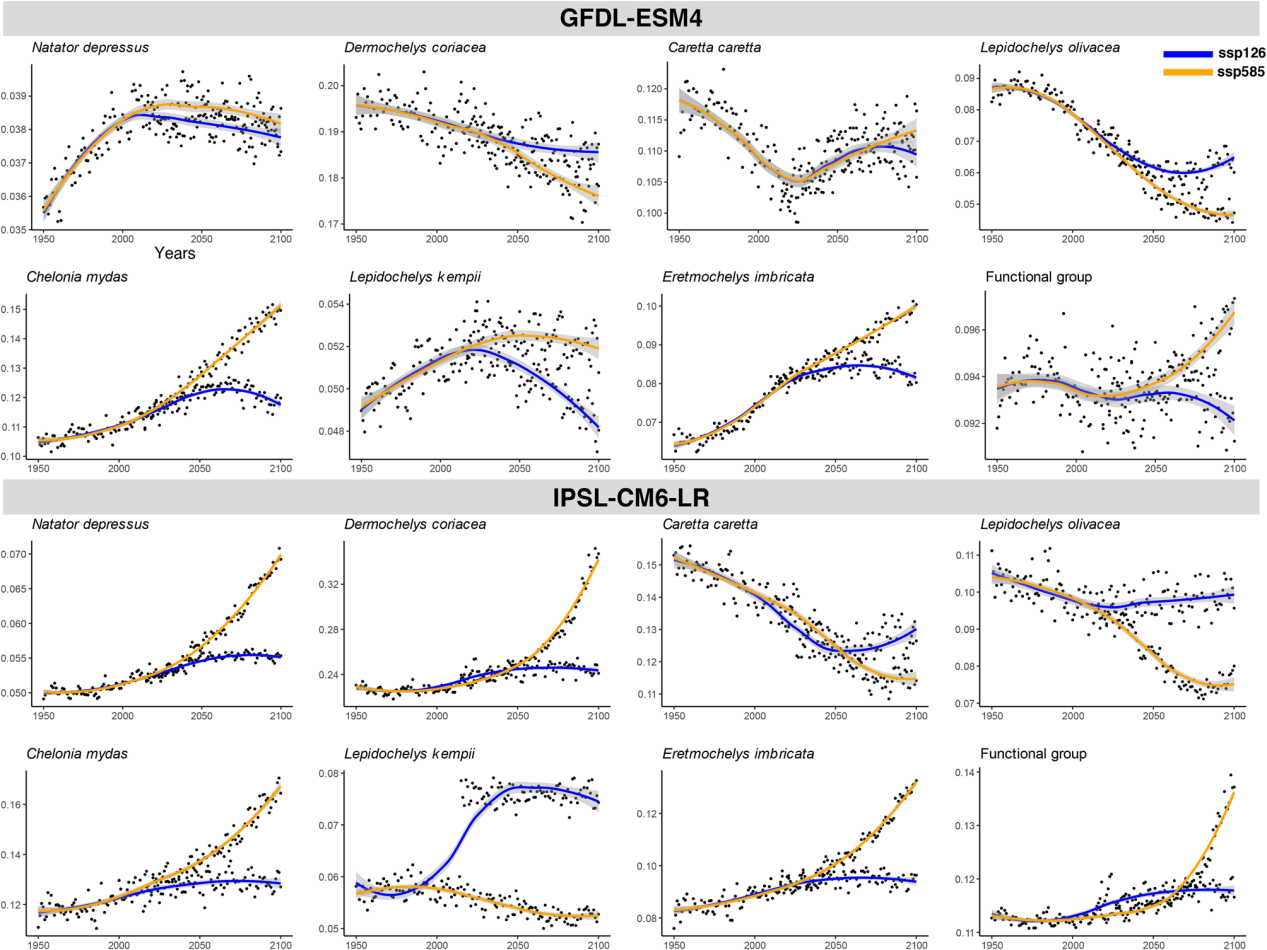
Changes over time in the mean probability of suitable habitat. The x-axis represents the years from 1950 to 2100, while the y-axis represents the mean probability for each year of the projected suitable habitat. The orange line represents the climate scenario SSP585, and the blue line represents the SSP126 climate scenario. Dots represent the mean probability calculated for each year.

Temporal trends in the mean probability of suitable habitat from 1950 to 2100 are shown for seven marine turtle species and their aggregated functional group under two climate scenarios, SSP126 and SSP585, using two ESMs: GFDL-ESM4 and IPSL-CM6A-LR (Fig. 4). Each panel displays annual mean values, with black dots representing yearly estimates and smoothed lines indicating trends under SSP126 (blue) and SSP585 (orange).

Under GFDL-ESM4, most species exhibit higher probabilities of suitable habitat under SSP585 compared to SSP126, especially in the latter half of the century. Species such as *C. mydas*, *L. kempii*, and *E. imbricata* show pronounced increases in suitability under SSP585, whereas *L. olivacea* shows a notable decline over time in both scenarios. In contrast, *D. coriacea* shows reduced suitability under SSP585 but relatively stable trends under SSP126. IPSL-CM6A-LR model projects more dramatic increases in suitable habitat under SSP585, particularly for *N. depressus*, *C. caretta*, and *C. mydas*. Some species, such as *L. olivacea*, show continuous declines under both scenarios. The functional group trends largely reflect the dominant patterns observed across species, with increasing probabilities under SSP585 and more stable or moderate changes under SSP126. Overall, SSP585 consistently predicts higher gains in suitable habitat, but with strong interspecific and model based variation in both direction and magnitude of change.

To assess long-term changes in habitat suitability, we calculated the mean probability of suitable habitat for each species and their functional group during the historical period (1950–2014) and compared it to the mean for the final decade of projections (2090–2099). The resulting percentage change is reported under two Earth System Models (GFDL-ESM4 and IPSL-CM6A-LR) and two climate scenarios (SSP126 and SSP585) (Table 2).

Under the GFDL-ESM4 model, species responses vary substantially. For example, *E. imbricata* shows the greatest positive change, with a 45.59 % increase under SSP585, while *L. olivacea* exhibits the most pronounced decline, with a 44.51 % reduction under the same scenario. Most species experience greater increases under SSP585 than SSP126, except for *C. caretta*, which shows slight improvement under SSP585 (1.97 %) relative to a stronger decline under SSP126 (4.83 %).

In contrast, the IPSL-CM6A-LR model projects consistently stronger positive trends under SSP585 for most species. Notably, *E. imbricata*, *D. coriacea*, and *N. depressus* show large projected increases in suitable habitat, exceeding 47 %, with *E. imbricata* reaching 62.18 %. However, not all species benefit; *C. caretta* and *L. olivacea* show declines under both scenarios, with the strongest losses under SSP585.

**Table 2:** Percentage (%) of increase or decrease of the suitable habitat’s mean probability between the historical suitable habitat (1950–2014) and the last ten years of the future suitable habitat’s projections (2089–2099).

Species	GFDL-ESM4		IPSL-CM6A-LR	
	SSP126	SSP585	SSP126	SSP585
*Natator depressus*	1.81	2.21	12.88	47.14
*Dermochelys coriacea*	$-2.62$	$-6.97$	10.53	57.12
*Caretta caretta*	$-4.83$	$-1.97$	$-14.72$	$-23.25$
*Lepidochelys olivacea*	$-24.31$	$-44.51$	$-1.45$	$-29.35$
*Chelonia mydas*	11.70	40.11	9.17	41.63
*Lepidochelys kempii*	$-2.85$	4.45	36.68	$-9.31$
*Eretmochelys imbricata*	21.38	45.59	11.74	62.18
Functional group	$-0.80$	3.51	26.58	43.98

#### Functional group predictions

**Fig. 5:**
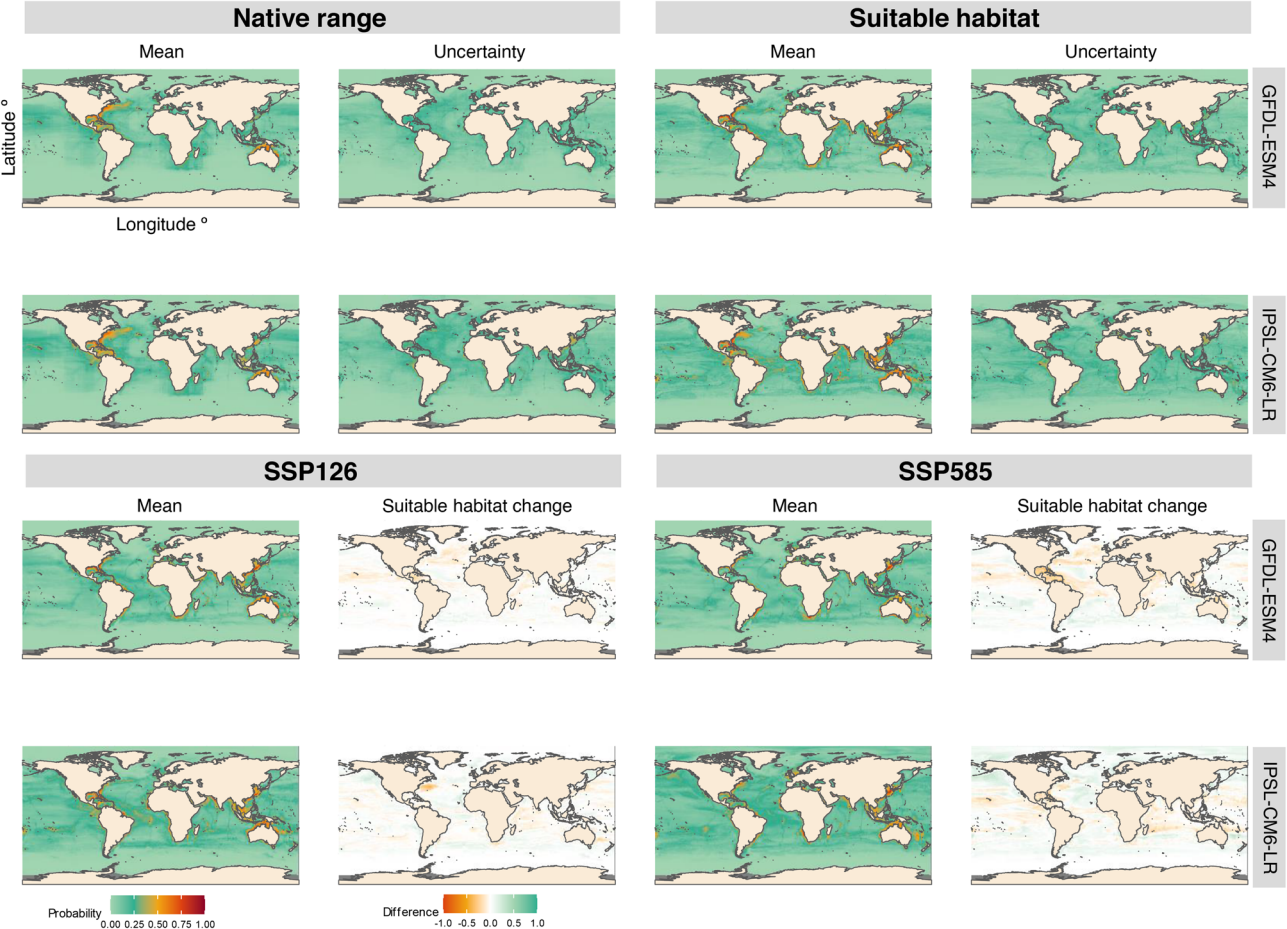
Functional group results of the native ranges and suitable habitats (1950–2014) and future suitable habitats (2015–2100) are provided. Rows one and two represent the spatial predictions for the current distribution, while the third and fourth rows depict the predictions for the last ten years of projections (2089–2099), including the difference between the projections and the current suitable habitat. All of these are represented for both climate scenarios and ESMs.

The spatial distribution of the functional group’s native range, current suitable habitat (1950–2014), and future suitable habitat (2089–2099) is shown under two climate scenarios (SSP126 and SSP585) and two Earth System Models (GFDL-ESM4 and IPSL-CM6A-LR) (Fig. 5). Each row in the figure corresponds to a different model, and the outputs represent ensemble projections obtained by aggregating the results across all species, using the median probability value for both historical and future periods.

The maps display both the posterior mean probability of presence and the associated uncertainty for native ranges and current suitable habitats (Fig. 5). Uncertainty is computed as the range between the 97.5% and 2.5% percentiles of the posterior predictive distribution. Across both models, uncertainty remains relatively narrow, with the lowest levels observed in the native range estimates, likely due to the strong constraint imposed by occurrence data (Fig. 5). The highest probabilities for the native range are concentrated along the eastern coast of the United States and the northern coast of Australia, while suitable habitat projections highlight a broader set of coastal regions with favorable environmental conditions (Fig. 5). In contrast, the lowest probabilities across all habitat types and models are consistently found near the polar regions (Fig. 5).

Future conditions are also shown for the period 2089–2099, including both the projected mean probabilities and the spatial differences relative to the current suitable habitat (Fig. 5). These maps highlight areas where habitat suitability is expected to increase or decrease under the two climate scenarios, SSP126 and SSP585. The results reveal clear differences between native range and projected suitable habitat, and indicate a poleward shift in suitable areas. These changes are more pronounced under the SSP585 scenario, which shows broader expansions of suitable habitat, especially across temperate regions (Fig. 5).

Complementing the spatial projections, Fig. 4 and Table 2 summarize the temporal trends and quantify the overall percentage change in habitat suitability from the historical period to the end of the 21st century. Under the SSP585 scenario simulated by IPSL-CM6A-LR, the mean probability of suitable habitat increases by 43.98% in the final decade of projections compared to the historical baseline. In contrast, projections from GFDL-ESM4 show smaller changes, with a 0.80% decrease under SSP126 and a slight 3.5% increase under SSP585.

## Discussion

The results from this study underscore the strength of Bayesian Additive Regression Trees (BART) as a robust and versatile tool to model species distributions on a global scale. The simulations and the marine turtles case study not only validate the predictive capacity of BART but also illustrate its ability to adapt to complex spatio-temporal patterns in diverse ecological scenarios. As a flexible, non-parametric machine learning approach, BART can handle non-linear relationships and integrate multiple environmental variables while avoiding overfitting through its Bayesian framework.

According to the simulation results, BART consistently provided accurate predictions for both cosmopolitan and persistent species. All models included in this simulation study (GAMs, MaxEnt and BART) were able to capture key patterns in spatial and temporal distributions, maintaining high sensitivity, specificity, and accuracy throughout the simulated periods. However, BART outperformed traditional methods such as GAM and MaxEnt, especially under conditions where true absence data were not available and pseudo-absences had to be generated. Therefore, our results show that BART, when combined with simulation-based evaluations, represents a strong alternative for modeling species distributions, particularly under conditions of data uncertainty and limited absence information.

Regarding the marine turtles case study, our results highlight that climate change stands as a significant threat to many marine species^57^. Among them, marine turtles are commonly considered susceptible to the impacts of climate change due to the important role of temperature in their life cycle^57,58^. In 2009, the IUCN Red List categorized most of the marine turtles species as vulnerable, endangered, or critically endangered^59–65^. Therefore, preserving marine turtle populations under new climate change conditions demands global actions to reduce its impact and bolster turtle resilience^57^.

Our study successfully tested the capability of global SDMs to investigate the global distribution of marine turtles and project future potential habitats under different scenarios of climate change. In fact, our results highlight the heterogeneity distribution of such a diverse group and shows the divergence in future projections according to specific species. While some species are likely to face important challenges in the future and may experience declines in available suitable habitats, others are projected to expand their potential habitats. These results highlight the need for management actions to be tailored to specific species and regions.

Therefore, based on the future suitable habitats obtained in our research, future studies could focus on analyzing the ability of different species of marine turtles to reach this new potential habitat^66^. It’s important to consider that even if a habitat is suitable in terms of environmental conditions, factors such as proximity or other non-environmental drivers may prevent the species from reaching that new potential habitat^67–69^.

Model results also confirm the important role of sea temperature to drive species distributions, and specifically the distribution of marine turtles. The increase in sea surface temperatures due to global warming can have various impacts on marine turtles, affecting their habitats, food sources, reproductive patterns, and overall survival^70^. For example, rising sea levels and increased temperatures can lead to the loss of nesting beaches for marine turtles. Coastal erosion can destroy nesting sites, making it challenging for turtles to find suitable areas to lay their eggs^71^. In the past decade, there has been an observed rise in sporadic nesting occurrences of sea turtles^72^, notably linked to unusual increases in Sea Surface Temperature (SST)^73^.

In the current context of conservation and management of marine turtle, another important threat to these species today is bycatch in fishing gears^74,75^. As mentioned, our results can be used to identify current hotspots of species richness of marine turtles and be used to minimize fishing practices in those areas with higher risk of by catch. Consequently, an expansion of suitable areas for marine turtles to specific areas should be done minimizing the risk of interactions with fishing gear. This brings to light the intricate balance between conservation efforts and the unintended consequences that may arise from increased suitable habitats intersecting with fishing activities.

Overall, forecast models such as the ones presented in the current study could help to inform conservation efforts of marine turtles, and to minimize incidental capture in fishing gear, potentially through the establishment of protected marine areas. In fact,^76^ proposed the use of Regional Marine Turtle Management Units (RMUs) as a framework for prioritizing conservation across multiple scales of sea turtles. However, this RMU overview could completely change due to climate change. While expanding suitable areas for marine turtles is crucial for their conservation, it necessitates a comprehensive understanding of the intricate interplay between habitat availability, fishing activities, and the broader ecosystem dynamics. Integrating these complexities into conservation models and strategies is imperative to ensure the long-term survival of marine turtle populations.

Although we acknowledge that BART is a useful tool for solving ecological issues, our study also has some limitations. One main concern is the uncertainty linked to the data we used. We relied on the GBIF database, which may have a large amount of uncertainty within its observations. Despite that, we have followed standard procedures to clean up and improve the quality of the data. Similarly, environmental drivers could involve significant uncertainty, particularly in future projections. To partially account for some of the uncertainty, we utilized two different Earth System Model (ESM) outputs, ensuring that we do not rely only on a single set of drivers. Indeed, for some species of marine turtles, GFDL-ESM4 and IPSL-CM6-LR lead to different results in terms of future potential habitats. This raises the need of considering the uncertainty related to ESMs when we use environmental drivers as inputs, which has been already observed in previous studies^77^.

Another limitation is on how we generated pseudo-absences. Since we lack absence data, we had to create pseudo-absences. We have tried to make this in a way that does not heavily impact the results, using random generation and equal amounts of absences and presences. Furthermore, to address these concerns, we conducted a simulation study to better assess the performance of BART. This helped us to have a more reliable understanding of the tool’s capabilities, particularly in combination with a rigorous case study. In addition, while the BART model does not explicitly incorporate spatial autocorrelation, recent extensions of the method have begun to address this limitation, and exploring such approaches represents an important direction for future research^78^.

Despite the valuable utility of SDMs in estimating distribution changes over time, there is an ongoing need to enhance these models^79^. Combining complementary models can produce better results, providing a more comprehensive understanding of species behavior^80^. For example, data from databases such as GBIF often come from various sources, making the use of different SDMs depending on the type of data a key ongoing topic in SDM research^81–84^. Moreover, it is important to note that our predictions do not account for changes in ecological relationships, such as prey-predator dynamics or changes in key demersal habitats, nor other crucial factors like fisheries mortality. Changes in sea temperatures can alter the distribution and abundance of marine turtle prey, such as jellyfish, crustaceans, and sea grasses. This can impact the feeding habits of turtles and affect their growth and health but our results can only capture this implicitly. In addition, marine turtles rely on coral reefs for food and shelter. Increased sea temperatures can lead to coral bleaching events, which reduce the quality and availability of habitat for turtles and their prey. This is the case for *Eretmochelys imbricata*, which exhibits strong associations with coral reef ecosystems, feeding predominantly on sponges^85^. Hence, their distribution might be more closely linked to food availability than to other environmental factors. This underscores the complexity of factors governing the distribution and habitat preferences of marine turtles, suggesting that conservation strategies should consider specific dietary needs and habitat dependencies of individual species. Hence, it’s relevant to integrate models, such as SDM and MEMs, to account for these additional relationships^80,86^. As such, our findings have significant potential value for parameterizing MEMs in order to improve the overall accuracy of predicted spatial-temporal species distributions of marine species, such as marine turtles, globally.

Due to the potential use of global SDMs, it is crucial to continue developing tools that allow us to assess the past, present, and future status of marine species, such as marine turtles^87^. In this context, the results obtained in this study highlight the ability of machine learning models such as BART to predict changes in the current and future habitats of marine species, making these models a valuable approach for assessing management and conservation efforts^37^. Our study shows how BART can be a reliable tool for predicting both current and future habitats of marine turtles on a global scale. We anticipate significant developments in both current and future applications of global SDMs approaches.

## Methods

This section includes the methodological details of our case study and the simulation study. The entire analysis was conducted using the R programming language^88^ and all code is available in GitHub.

### Simulation

We conducted a simulation designed to corroborate the predictive capabilities of BART in both spatial and temporal dimensions. This simulation involved two specific scenarios: (1) simulating a cosmopolitan species dispersed across the entire domain, and (2) simulating a persistent species with consistent spatial and temporal patterns. Additionally, we included an analysis to assess how sensitive the BART model is to the generation of pseudo-absences, further ensuring the robustness of its predictions. To contextualize the performance of BART, we also compared its results with those obtained using traditional species distribution modeling approaches, specifically MaxEnt and Generalized Additive Models (GAMs). Through this process, our objective was to provide further evidence supporting the reliability of BART to accurately predict the dynamics of species distribution over space and time.

Simulation allowed us to replicate the behavior of a random variable in both space and time under controlled conditions, such as the probability of presence of a species population. Therefore, the first consideration in simulation is understanding the factors influencing our variable of interest and developing a model that accounts for its nature. Typically, we lack information about the entire population and work with a sample instead. In such cases, we propose a model and make inferences about its parameters to obtain representative insights into the population. However, when simulating the entire population, we have knowledge of the parameters, enabling us to assess the accuracy of our model estimates^89^.

For a more detail explanation and figures of the simulation process refer to the following vignettes.

#### Spatio-temporal occurrence simulation scenarios

The probability of the presence of a given target species is commonly influenced by various external factors (e.g., environmental, anthropogenic, etc.) as well as spatially structured biological processes (e.g., predation, competition, etc.). Moreover, Ref.^90^ argue that all species, in one way or another, exhibit spatial structure. However, considering all the factors that affect the probability distribution of a target species in the modeling is practically impossible. For this reason, we have simplified the reality of our response variables taking into account two environmental variables (temperature and bathymetry) as essential drivers to explain distributions, a temporal dependence over the years, and a spatial-temporal effect related to species movement and dispersal. This selection was made considering that temperature and bathymetry typically play a key role in the spatial and temporal distribution of marine species^91^. Additionally, Ref.^92^ discuss how incorporating a spatial effect can enhance prediction accuracy and mitigate the impact of variables not considered in the modeling. Hence, the simulation models for the different scenarios (cosmopolitan and persistent species) are formulated as follows:

1. **Cosmopolitan species**
  1 $$\begin{aligned} \begin{aligned} Y(s,t)&\sim \text {Bernoulli}(\pi (s,t)), \\ \text {logit}(\pi (s,t))&= \beta _{0} + f_{1}(t) + f_{2}(\text {X}_{1}(s)) + \beta _{1}X_{2}(s,t) + U(s,t), \end{aligned} \end{aligned}$$
  where, the response *Y*(*s*, *t*) represents the occurrence (presence/absence) of the cosmopolitan species at time *t* in the location *s* following a Bernoulli distribution with parameter $\pi (s,t)$; $\pi (s,t)$ is linked to the predictor by the logit link function; $\beta _{0}$ is the intercept; $f_{1}(t)$ stands for the temporal trend in the year *t*; $f_{2}(\cdot )$ is a deterministic function for the bathymetry ($X_{1}(s)$); and $\beta _{1}$ is the parameter associated to the temperature ($X_{2}(s,t)$). Lastly, *U*(*s*, *t*) refers to the spatio-temporal structure.
2. **Persistent species**
  2 $$\begin{aligned} \begin{aligned} Z(s,t)&\sim \text {Bernoulli}(\pi (s,t)), \\ \text {logit}(\pi (s,t))&= \beta _{0} + f_{1}(t) + \beta _{1}X_{1}(s) + \beta _{2}X_{2}(s,t) + U(s,t), \end{aligned} \end{aligned}$$
  where the response *Z*(*s*, *t*) represents now the occurrence (presence/absence) of the persistent species at time *t* in the location *s* following a Bernoulli distribution with parameter $\pi (s,t)$; $\beta _{1}$ is a fixed effect for the bathymetry $X_{1}(s)$; and $\beta _{2}$ is the parameter associated to the temperature $X_{2}(s,t)$; and the remaining terms are those in 1.

With the model structure determined to start simulating the occurrence data of both scenarios ($Y(s,t) \, \text {and} \, Z(s,t)$), some explanation is warranted to describe how to perform these simulations, in particular, how to deal with each one of the terms included in the predictors in Eqs. (1) and (2). First, the spatio-temporal structure is simulated as a Gaussian Markov Random Field (GMRF) correlated with an autoregressive *AR*(1) model with parameter of autocorrelation $\rho _{sp}$^93^. Secondly, we simulate species-specific depth preferences. Particularly, for the bathymetry covariate, a range between 0-800 meters was simulated, with a non-linear effect for the cosmopolitan species scenario $f(X_{1}(s))$ and a linear effect for the persistent species scenario $\beta _{1}X_{1}(s)$. Last, for the temporal trend *f*(*t*), changes in the probability values over time are included by simulating a vector of values from an autoregressive model of order 1 with parameter of autocorrelation $\rho _{t}$.

Once the predictor terms have been obtained, the occurrence of both species (*Y*(*s*, *t*) and *Z*(*s*, *t*)) has to be determined by using a Bernoulli distribution. Then, once we have obtained the simulated presence/absence data that will be fitted with the BART model (Eq. 3), we need to perform several random samplings of each simulation. In this case, we conducted 50 samplings for each simulation scenario, allowing us to replicate the simulation and ensure the robustness of the analysis. Additionally, beyond fitting the model with true absence data, we also generated pseudo-absence data by randomly selecting points of presence and absence in the sampling process.This process of generating pseudo-absences was also repeated 50 times, giving us a total of 100 BART models to fit per species (50 with true absences and 50 with pseudo-absences). This approach enabled us to compare the performance of the models using true absence data versus pseudo-absence data, providing a more comprehensive understanding of the model’s predictive capabilities under different scenarios. For model validation, we calculated three commonly used measures: sensitivity, specificity, and accuracy. To achieve this, we compared the estimated values (whether they indicate presence or absence) with the actual simulated presence or absence data. This process allowed us to determine how effectively our model assigns the correct status of presence or absence in relation to the simulated ground truth.

### Case study

The focus of this study is to estimate and predict the probability of presence over space and time for the marine turtles functional group (refer to Table S1 of supplementary material) for biological information about the species). In order to achieve our goal, a series of steps were carried out. First, presence data of each marine turtle species and environmental variables potentially driving their distribution were extracted and cleaned. Then, the BART model was implemented using the collected data of individual species. Last, the different results were validated and compared.

#### Extraction and cleaning of the data

Presence-only data of a species are one of the most widely used datasets in the context of SDMs due to their accessibility at different scales^94–96^. For our study, which aims to predict using a global perspective, we obtained data from the Global Biodiversity Information Facility (GBIF) using the rgbif package in R^97,98^. All the DOIs with the downloaded raw data for each species are available in the supplementary material section 1 Marine turtles information and study workflow.

The presence data for the seven species of marine turtles currently occurring in the marine environment were processed by eliminating repeated and terrestrial locations. We excluded terrestrial locations because we were only interested in predicting distribution in the oceans. However, it’s worth mentioning that female marine turtles spend part of their life cycle on land. We also employed the CoordinateCleaner package in R to remove presences with significant uncertainty^99^. BART requires both presence and absence data to operate correctly. Due to the lack of available absence data for statistical modeling using a Bernouilli distribution, we randomly generated pseudo-absences equal to the number of presences for each species^100^.

Furthermore, we incorporated global spatial time series of varying environmental conditions obtained from The Inter-Sectoral Impact Model Intercomparison Project (ISIMIP)^54,55^ and Fish-MIP initiative (https://fish-mip.github.io/). We drove our model using outputs from two different Earth System Models (ESMs) of the Coupled Model Intercomparison Project Phase 6 (CMIP6): GFDL-ESM4 and IPSL-CM6A-LR^101^. These models were built under prescribed scenarios for historic (1950–2014) and future (2015–2100) time periods^101^. Moreover, for both ESMs, we used two different Shared Socio-economic Pathway (SSP) climate scenarios: a more conservative one, SSP126, and a more pesimistic one, SSP585.

Among the various ESM variables available under ISIMIP, we selected SST (Sea Surface Temperature in degree Celsius), SSS (Sea Surface Salinity in PSU), LPHY (mole content of diatoms), O2 (mole concentration of dissolved molecular oxygen), DPHY (mole content of diazotrophs), and SPHY (mole content of picophytoplankton). It is worth noting that the last two variables were only available for GFDL-ESM4. Additionally, we included bathymetry as a static variable for all analysis. To prepare the variables for predictions, we standardized all the environmental variables. To standardize the variables, we applied the z-score transformation $X_{\text {std}} = \frac{X - \mu }{\sigma }$, where $X$ is the original variable, $\mu$ is the mean and $\sigma$ is the standard deviation calculated from the historical data. This standardization was applied to both historical and future layers to ensure comparability. However, for obtaining the functional responses, we utilized the non-standardized environmental variables to get the response curve in the real scale.

#### Modeling approach: BART

Regarding statistical modeling, BART models are based on a sum of regression trees. Reference^39^ provide an illustration of the formulation and representation of a single tree model, offering a comprehensive insight into the formulation underlying these models. Essentially, regression trees are algorithms meant for modeling and prediction in machine learning^102,103^. The formulation of a regression tree *g* could be defined in terms of two components: (1) T a set of decision rules and nodes, and (2) $M={\mu _{1},...,\mu _{b}}$ a set of parameter values associated to each terminal node of T. Then, *g*(*X*; *T*, *M*) is the function that assigns a value to the *b* parameters ($M={\mu _{1},...,\mu _{b}}$) according to the covariates (*X*) added to the tree model.

The main problem with regression trees is that they tend to overfit, as they can split the space until they get one parameter per datum^39^. This overfitting may considerably bias predictions. To address this problem, approaches such as BART have been developed. Through the ensemble of decision trees and regularization using a priori distributions in the Bayesian context, BART methods reduce the overfitting without performing a cross validation for model parametrization^37,38^. In our study, we adopted the default prior distribution for BART, as the literature praises its strong performance with default parameters^39^.

In order to model the presences/pseudo-absences data, the statistical model applied in this work was as follows:

3 $$\begin{aligned} \begin{aligned} Y_{i} \sim Ber(\pi _{i}), \quad i = 1,...,n, \\ \phi ^{-1}(\pi _{i}) = \sum ^{m}_{j}g_{j}({\textbf {X}};T_{j},M_{j}), \end{aligned} \end{aligned}$$

where $Y_{i}$ represents the presence/pseudo-absence of species for observation *i*; $\pi _{i}$ is the parameter of interest linked to the predictor by a link function; $\phi ^{-1}$ denotes the standard normal cdf (probit link function); $g_{j}$ is the $j-$th ($j=1,\ldots ,m$) tree of the form $g_{j}({\textbf {X}};T_{j},M_{j})$, where *m* is the total number of trees, *X* is a vector of multiple covariates, $T_{j}$ represents a binary tree structure consisting of a set of interior node decision rules and a set of terminal nodes, and $M_{j} = \{\mu _{j},...,\mu _{jb}\}$ denotes a set of parameter values associated with each of the $b_{j}$ terminal nodes of $T_{j}$.

In the binary classification model implemented using Bayesian Additive Regression Trees (BART) via the dbarts package^104,105^, prior distributions are used to enforce regularization and prevent overfitting. Tree structures are governed by a depth-dependent prior of the form $P(\text {split at depth} d) = \alpha (1 + d)^{-\beta }$, with default values $\alpha = 0.95$ and $\beta = 2$, encouraging shallow and simple trees. The values assigned to terminal nodes (i.e., individual tree predictions) follow a normal prior $\mathscr {N}(0, \sigma _\mu ^2)$, where $\sigma _\mu = \frac{3.0}{k \sqrt{m}}$, with $k$ as a regularization parameter and $m$ representing the total number of trees. In the classification setting, BART uses a probit model where the latent error term is fixed as $\epsilon \sim \mathscr {N}(0, 1)$, ensuring model identifiability and eliminating the need to estimate a residual variance.

Furthermore, a differentiation was established between two types of models: (1) native ranges, which refer to the areas where the species is known to have occurred historically and it is likely currently present; and (2) suitable habitats, which are understood as potential habitats where conditions are suitable for the target species. The reason for this differentiation is that certain areas may be considered potential habitats, but due to other factors such as geographic barriers or physical distances, the species has never been observed or is not present in those areas. Therefore, the main difference when modeling these two distributions is that for suitable habitats (2) the ${\textbf {X}}$ vector of covariates only includes environmental variables, while for native ranges (1) the ${\textbf {X}}$ vector of covariates also incorporates the coordinates of historical observations to account for realistic or plausible spatial variability in the model.

After inferring the model parameters, space and time predictions were carried out for the historical period (1950–2014) and for future projections (2015–2100) using two different ESMs (GFDL-ESM4 and IPSL-CM6A-LR) and climate change scenarios (SSP126 and SSP585). Hence, we generated the historical (1950-2014) and future (2015–2100) projections by year using the suitable habitat model. Consequently, the predictions provide insights into the future areas where environmental conditions will be optimal for the seven marine turtle species. In contrast, we generated two different aggregated historical distributions in space: one using the native range model and the other using the suitable habitat model. For these aggregated historical spatial distributions, we employed the mean of the environmental variables (see Figure S1 in the supplementary material). Moreover, to interpret the influence of predictors on species distribution, we computed functional responses using partial dependence plots, which show the marginal effect of individual variables on the predicted probability of presence. Variable importance was assessed using a permutation-based approach, which measures the increase in prediction error when the values of a single predictor are randomly permuted.

Given the complexity of estimating species distributions in changing environments, we employed Bayesian Additive Regression Trees (BART) to address the challenges of modeling non-linear relationships between species and their environment. BART offers flexibility and is particularly suitable for handling large datasets with multivariate covariates, a common feature in climate change-related projections. Since our study focuses on suitable habitat models, BART is a particularly useful tool to account for uncertainty in these types of projections. Additionally, we recognize that there are multiple established approaches to addressing the challenges of SDMs/ENMs, including several based on machine learning (ML). However, we believe that the use of ML in our study complements traditional approaches, as it allows for handling complex non-linear relationships between environmental variables and species distributions, as well as the ability to integrate large volumes of data in a flexible manner. In this sense, we are not proposing to replace traditional methods such as MaxEnt, Generalized Linear Models (GLMs), or Generalized Additive Models (GAMs), but rather to expand the predictive capabilities of species distribution models by highlighting an additional tool. This approach may be particularly useful in scenarios involving large datasets and complex projections, such as those required to forecast under future climate change conditions.

#### Validation and comparison of predictions

For the validation of the models, we calculate several measures, distinguishing between two types of validations: an internal validation using a k-fold cross-validation method, and an external validation using new species distributions from GBIF that were not included in the models. Therefore, for internal validation, we applied the k-fold method to assess the performance of our model in the historical period. For external validation, we calculated these measures by comparing future projections of several years with actual observations that were not used in the fitting process.

For internal validation, we divide the data into k = 10 subsets to test the predictive capacity of BART. Therefore, we obtained a total of 10 different replicas. To analyze the results, we calculated error measures such as sensitivity, specificity, accuracy, and $F_{1}$ score. All calculated measures are based on the estimations of true positives, true negatives, false positives, and false negatives^106^. Furthermore, as our forecasting extended from 2015 to 2100, we were able to compare the model predictions from 2015 to 2023 with observed data from GBIF to evaluate the model’s performance in projecting the distribution. We compared the observed data with the predicted probability values and calculated error metrics. These metrics are essential when dealing with presence and absence data. To calculate all these metrics, we used a cutoff that was determined by maximizing Youden’s Index^107^. These metrics are defined as follows:

$$\text {SPC} = \frac{TN}{N}; \quad \text {SEN} = \frac{TP}{P}; \quad \text {ACC} = \frac{TP + TN}{P + N}; \quad \text {F}_1 = \frac{2 \times TP}{2 \times TP + FP + FN},$$

where $TP$ and $TN$ denote the number of true positives and true negatives, respectively; $FP$ and $FN$ represent false positives and false negatives; $P$ and $N$ refer to the total number of actual positives and negatives in the dataset. Specificity (SPC) measures the proportion of true negatives correctly identified, while sensitivity (SEN) quantifies the proportion of true positives correctly detected. Accuracy (ACC) indicates the overall proportion of correctly classified instances. The F_1_ score provides a harmonic mean between precision and recall, offering a balanced measure of model performance in the presence of class imbalance.

Finally, we compared the historical predictions (1950–2014) of each species with the last 10 years (2090–2099), excluding 2100, to assess potential future habitat changes. We exclude the last year of the series due to the potential bias in the ESMs models for this final year of projection. To quantify these changes, we calculated the difference between the predicted historical distribution and the projections for the last ten years. This allowed us to estimate the extent of potential habitat change based on future climate change scenarios. Likewise, we extracted the mean probability for each projected year from 1950 to 2100, allowing us to assess changes over time in the mean probability of potential suitable habitat for each species and for the entire functional group.

## Supplementary Information

Supplementary Information.

## Data Availability

The datasets generated and/or analysed during the current study are available in the Global Biodiversity Information Facility (GBIF) repository, https://www.gbif.org/es/. DOIs are available in the supplementary material.
