# Supplementary material for: Machine learning applied to global scale species distribution models
Source: Sci Rep. 2025 Oct 27;15:37534. doi: 10.1038/s41598-025-20797-x (PMC12559289; doi:10.1038/s41598-025-20797-x)
Supplement: Supplementary file 1 — Supplementary Information. [file 41598_2025_20797_MOESM1_ESM.pdf]

Machine learning applied to global scale species distribution models

Alba Fuster-Alonso[1,7*], Jorge Mestre-Tomás[1], Jose Carlos Baez[2,3], Maria Grazia Pennino[4], Xavier Barber[5], Jose María Bellido[6], David Conesa[7], Antonio López-Quílez[7], Jeroen Steenbeek[8], Villy Christensen[9,8], and Marta Coll[1,8]

[1]Institute of Marine Sciences (ICM) - CSIC, Renewable Marine Resources Department, Barcelona, 08003, Spain.
[2]Spanish Institute of Oceanography (IEO) - CSIC, Oceanographic Center of Málaga, Fuengirola, 29640, Spain.
[3]Ibero-American Institute for Sustainable Development (IIDS), Autonomous University of Chile, Av. Alemania 1090, Temuco 4810101, Araucanía Region, Chile.
[4]Spanish Institute of Oceanography (IEO) - CSIC, Oceanographic Center of Madrid, C. Del Corazón de María, 8, 28002, Madrid, Spain.
[5]Center of Operations Research, Miguel Hernández University (UMH), Spain.
[6]Spanish Institute of Oceanography (IEO) - CSIC, Oceanographic Center of Murcia, San Pedro del Pinatar, Murcia, Spain.
[7]Department of Statistics and Operations Research (VaBar), University of Valencia (UV), Valencia, Spain.
[8]Ecopath International Initiative (EII), Barcelona, Spain.
[9]Institute for the Oceans and Fisheries, University of British Columbia, Canada.
[*]afuster@icm.csic.es

SUPPLEMENTARY MATERIAL

1 Marine turtles information and study workflow

Species	***IUCN Red List***	***Climate Zone***	***Distribution***
Natator depressus	Data Deficient	Tropical	Indo-West Pacific
Dermochelys coriacea	Vulnerable	Tropical	Circumglobal
Caretta caretta	Vulnerable	Tropical	Circumglobal
Lepidochelys olivacea	Vulnerable	Tropical	Indo-Pacific and Atlantic
Chelonia mydas	Endangered	Tropical	Circumglobal
Lepidochelys kempii	Critically Endangered	Tropical	Atlantic and Mediterranean
Eretmochelys imbricata	Critically Endangered	Tropical	Circumglobal

Table S1. Marine turtle species information from IUCN Red List and SeaLifeBase, including conservation status, climate zone, and distribution.

Species	*DOI*
Natator depressus	https://doi.org/10.15468/dl.wbweak
Dermochelys coriacea	https://doi.org/10.15468/dl.4ub6fn
Caretta caretta	https://doi.org/10.15468/dl.bvgx97
Lepidochelys olivacea	https://doi.org/10.15468/dl.bmhp5d
Chelonia mydas	https://doi.org/10.15468/dl.e3757n
Lepidochelys kempii	https://doi.org/10.15468/dl.5gzu4c
Eretmochelys imbricata	https://doi.org/10.15468/dl.8uw52a

Table S2. Digital Object Identifier (DOI) from GBIF for each marine turtle species.

2 Species predictions

For the species spatio-temporal predictions, we have two different outputs. Figures 1, 2 and 3 refer to the spatial current predictions (native ranges and suitable habitats) done in this work for the 7 species (1950-2014). While Figures 3, 4, and 5 refer to the suitable habitats obtain for those 7 species using future projections of environmental variables (2015-2100).

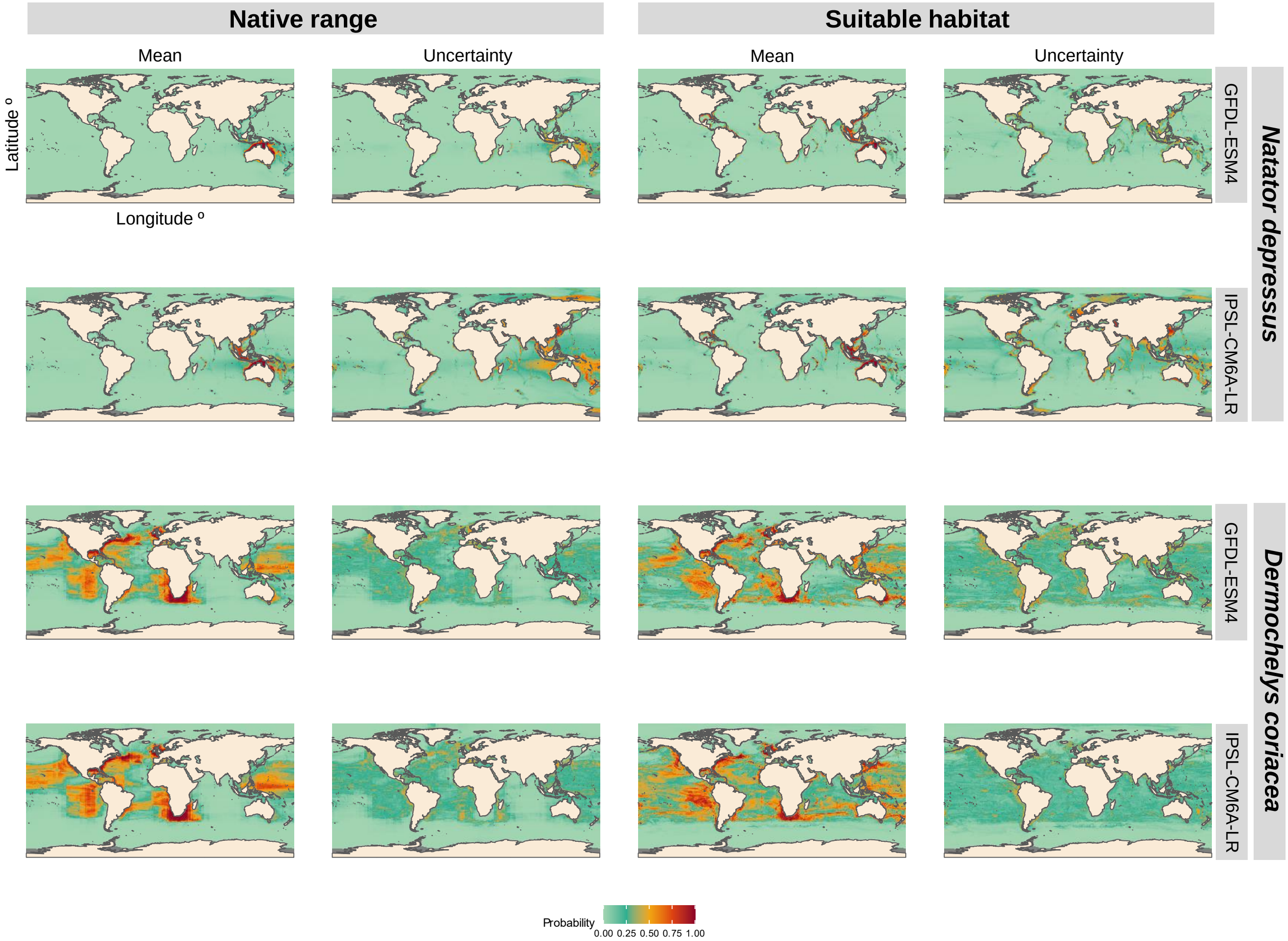

Figure S1. Maps depict the probability of presence for two species from 1950 to 2014, *Natator depressus* and *Dermochelys coriacea*. The first and second columns illustrate the native ranges (current distribution), while the third and fourth columns portray the suitable or potential habitats. The first and third rows correspond to the results for the GFDL-ESM4 model, while the second and fourth rows depict the results of IPSL-CM6ALR. We are presenting the mean posterior predictive distribution for both species, accompanied by uncertainty represented as the subtraction of quantiles 0.025 and 0.975.

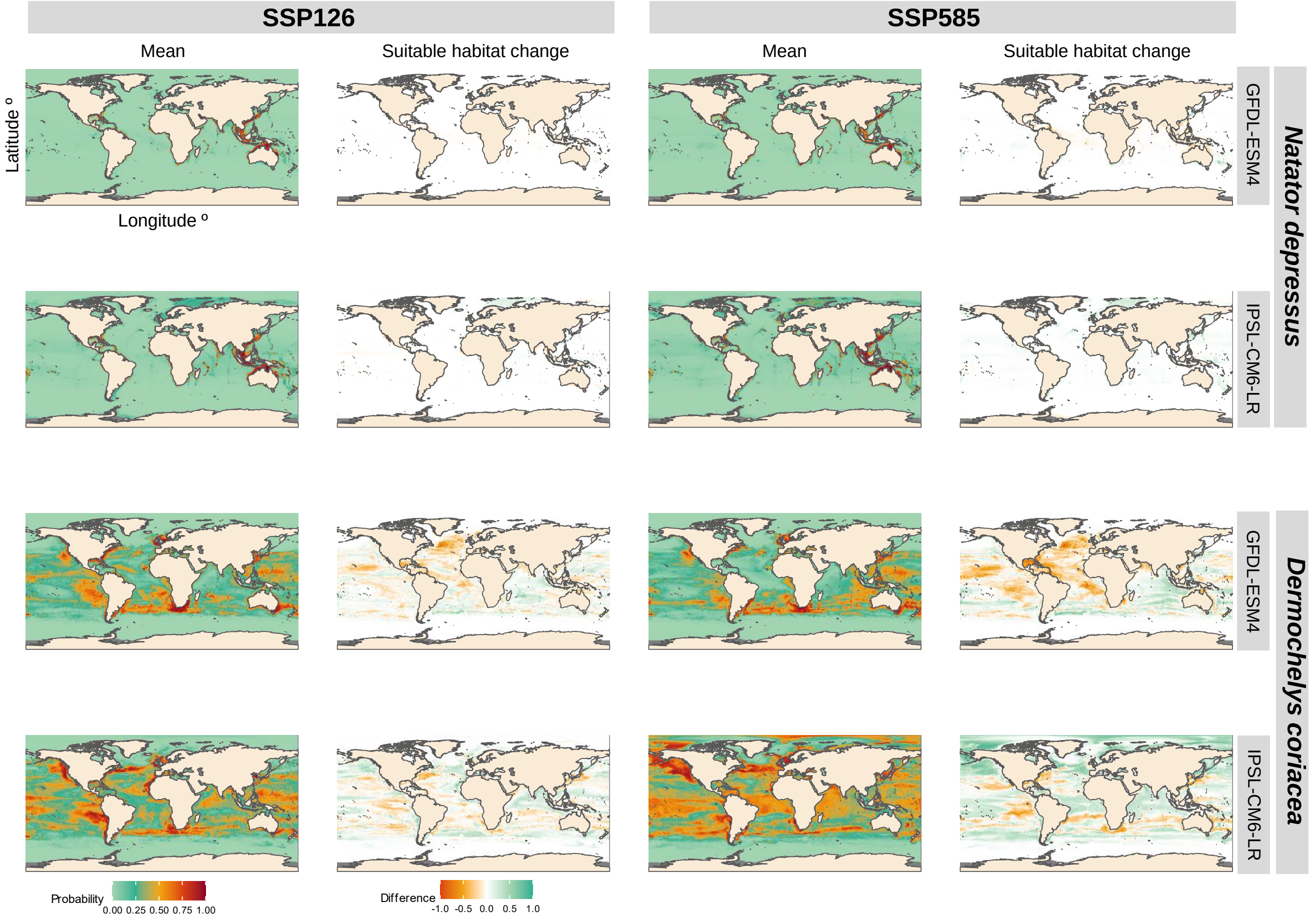

Figure S2. Maps representing the mean probability of presence from 2089 to 2099 for *Natator depressus* and *Dermochelys coriacea*, along with the difference between the historical suitable habitat (Figure 1) and the projections for the last 10 years (2089-2099). We have calculated the difference for both climate change scenarios, SSP126 and SSP585, and also for both Earth System Models (GFDL-ESM4 and IPSL-CM6A-LR).

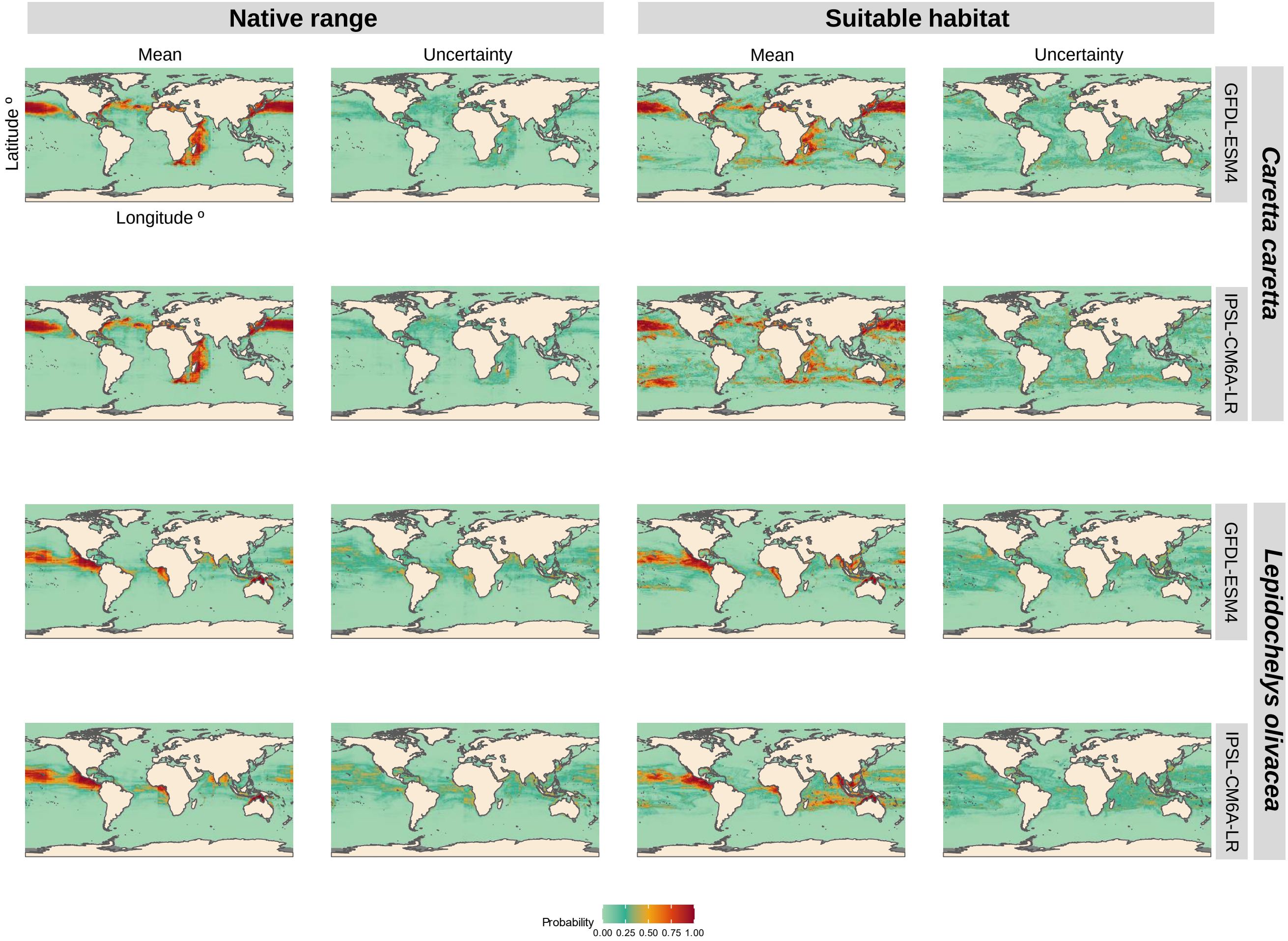

Figure S3. Maps depict the probability of presence for two species from 1950 to 2014, *Caretta caretta* and *Lepidochelys olivacea*. The first and second columns illustrate the native ranges (current distribution), while the third and fourth columns portray the suitable or potential habitats. The first and third rows correspond to the results for the GFDL-ESM4 model, while the second and fourth rows depict the results of IPSL-CM6ALR. We are presenting the mean posterior predictive distribution for both species, accompanied by uncertainty represented as the subtraction of quantiles 0.025 and 0.975.

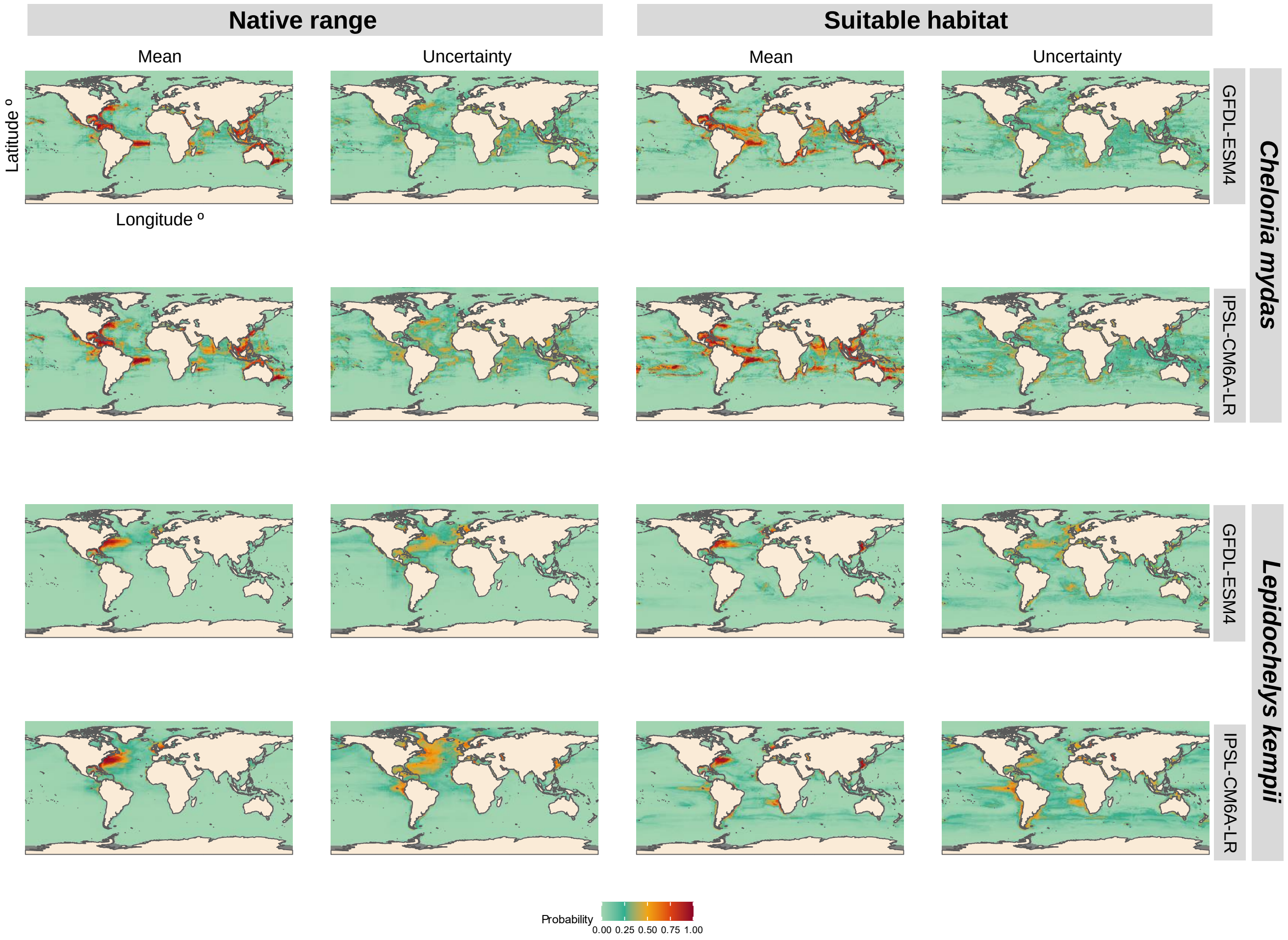

Figure S4. Maps depict the probability of presence for two species from 1950 to 2014, *Chelonia mydas* and *Lepidochelys kempii*. The first and second columns illustrate the native ranges (current distribution), while the third and fourth columns portray the suitable or potential habitats. The first and third rows correspond to the results for the GFDL-ESM4 model, while the second and fourth rows depict the results of IPSL-CM6ALR. We are presenting the mean posterior predictive distribution for both species, accompanied by uncertainty represented as the subtraction of quantiles 0.025 and 0.975.

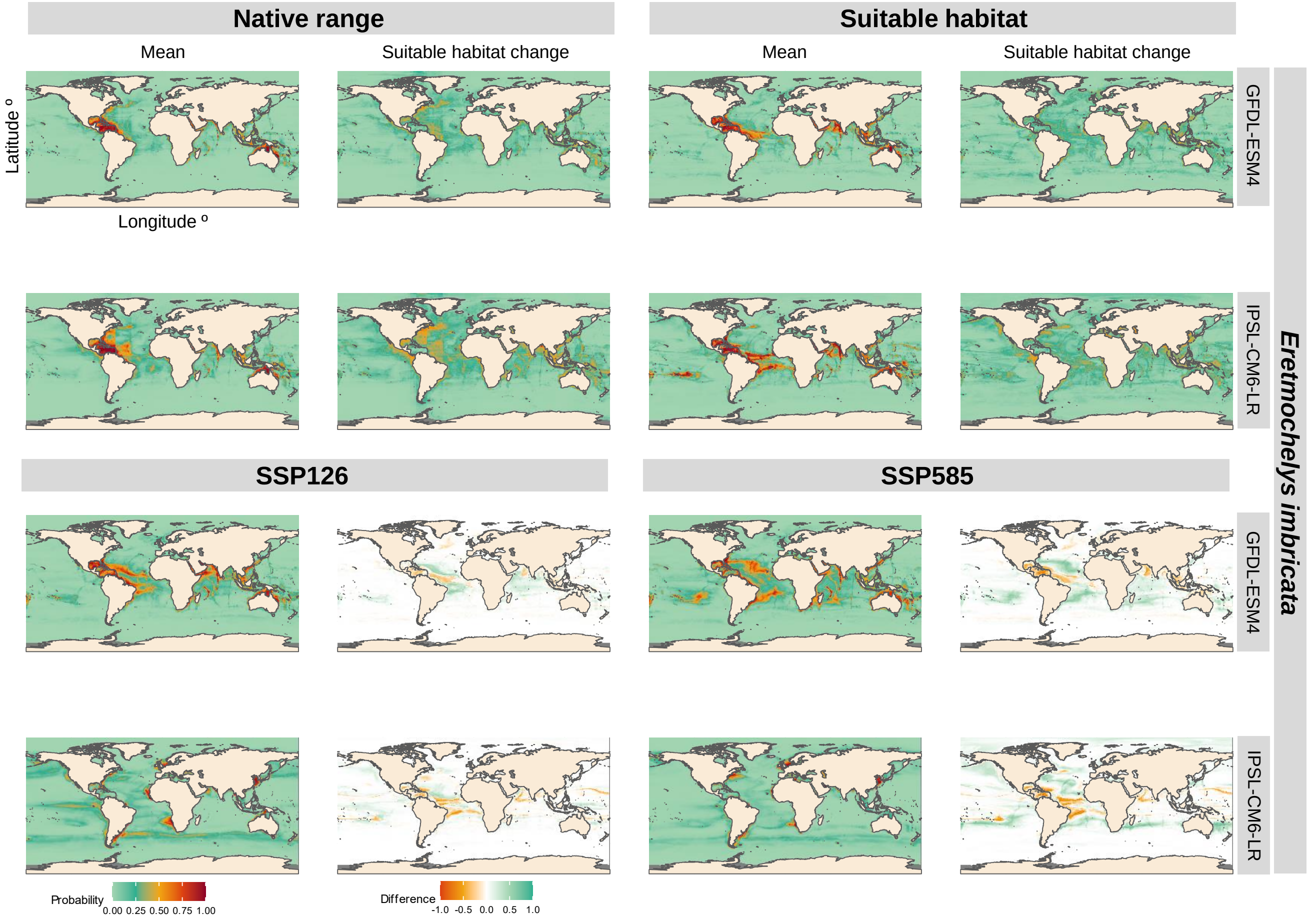

Figure S5. Maps depict the probability of presence for one species from 1950 to 2014 of *Eretmochelys imbricata*. The first and second columns and rows illustrate the native ranges, while the third and fourth columns and the first and second rows portray the suitable or potential habitats. We are presenting the mean posterior predictive distribution, accompanied by uncertainty represented as the subtraction of quantiles 0.025 and 0.975. Rows thrid and fourth represent the maps with the mean probability of presence from 2089 to 2099, along with the difference between the historical suitable habitat and the projections for the last 10 years (2089-2099).

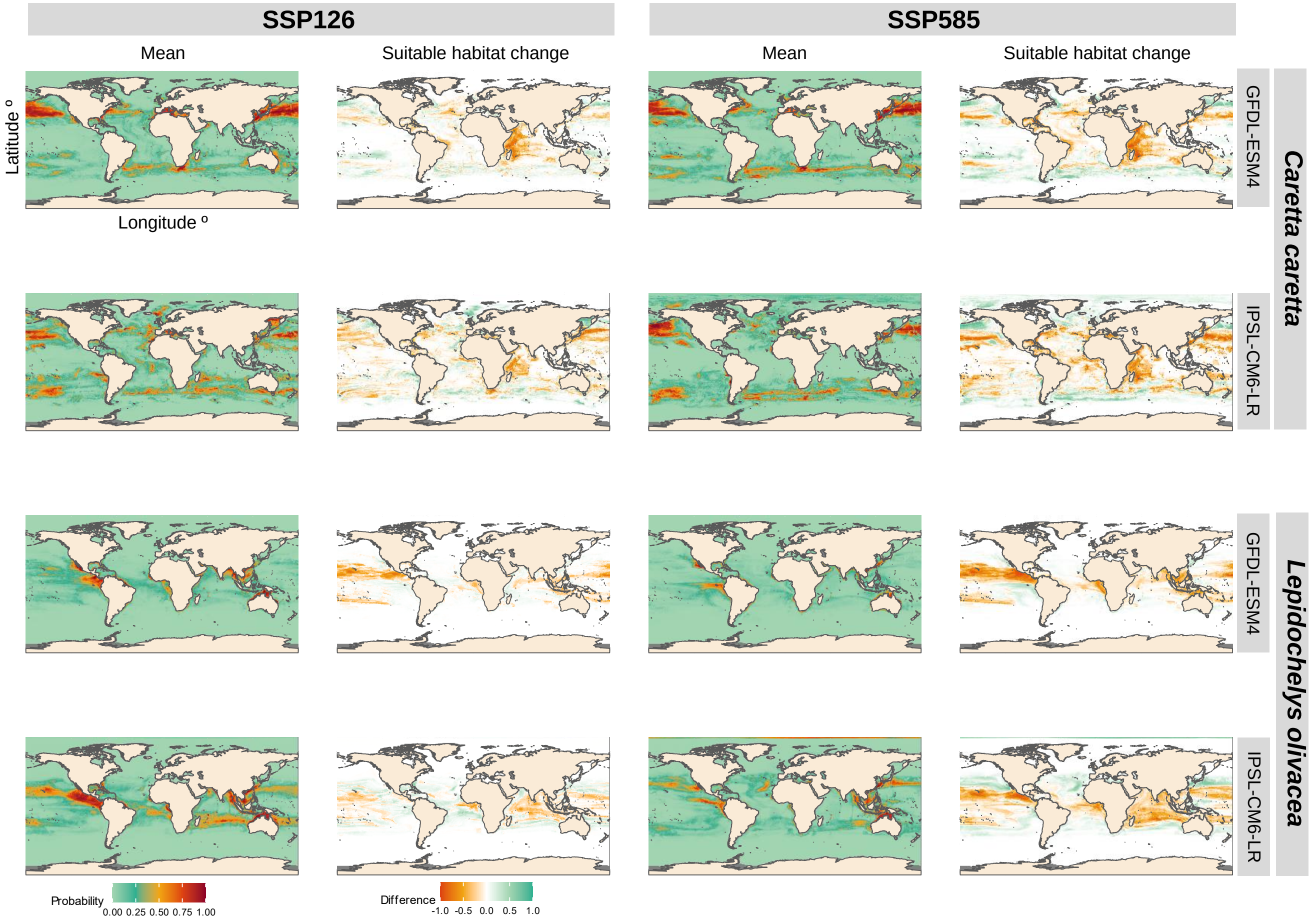

Figure S6. Maps representing the mean probability of presence from 2089 to 2099 for *Caretta caretta* and *Lepidochelys olivacea*, along with the difference between the historical suitable habitat and the projections for the last 10 years (2089-2099). We have calculated the difference for both climate change scenarios, ssp126 and ssp585, and also for both Earth System Models (GFDL-ESM4 and IPSL-CM6A-LR).

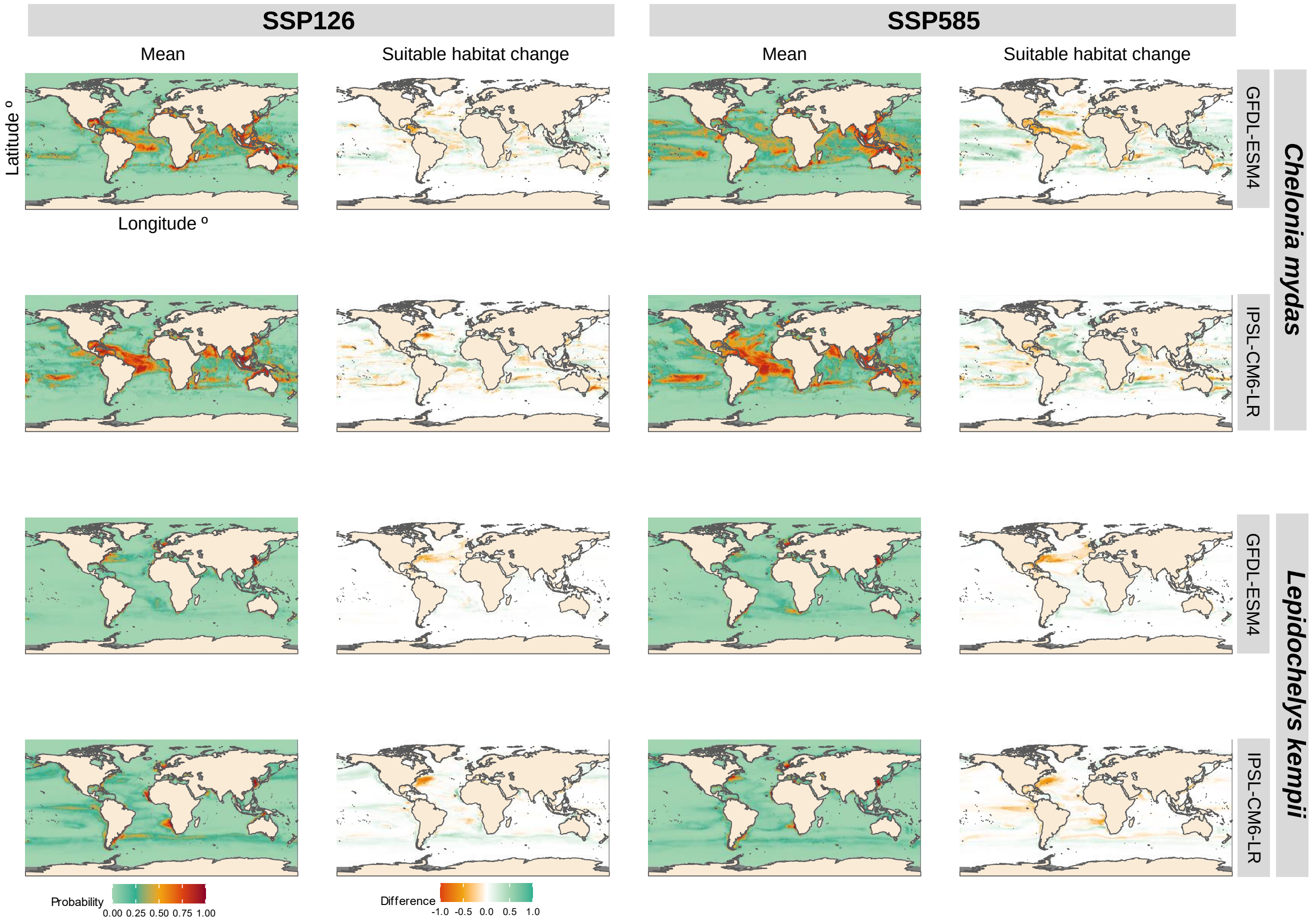

Figure S7. Maps representing the mean probability of presence from 2089 to 2099 for *Chelonia mydas* and *Lepidochelys kempii*, along with the difference between the historical suitable habitat and the projections for the last 10 years (2089-2099). We have calculated the difference for both climate change scenarios, ssp126 and ssp585, and also for both Earth System Models (GFDL-ESM4 and IPSL-CM6A-LR).

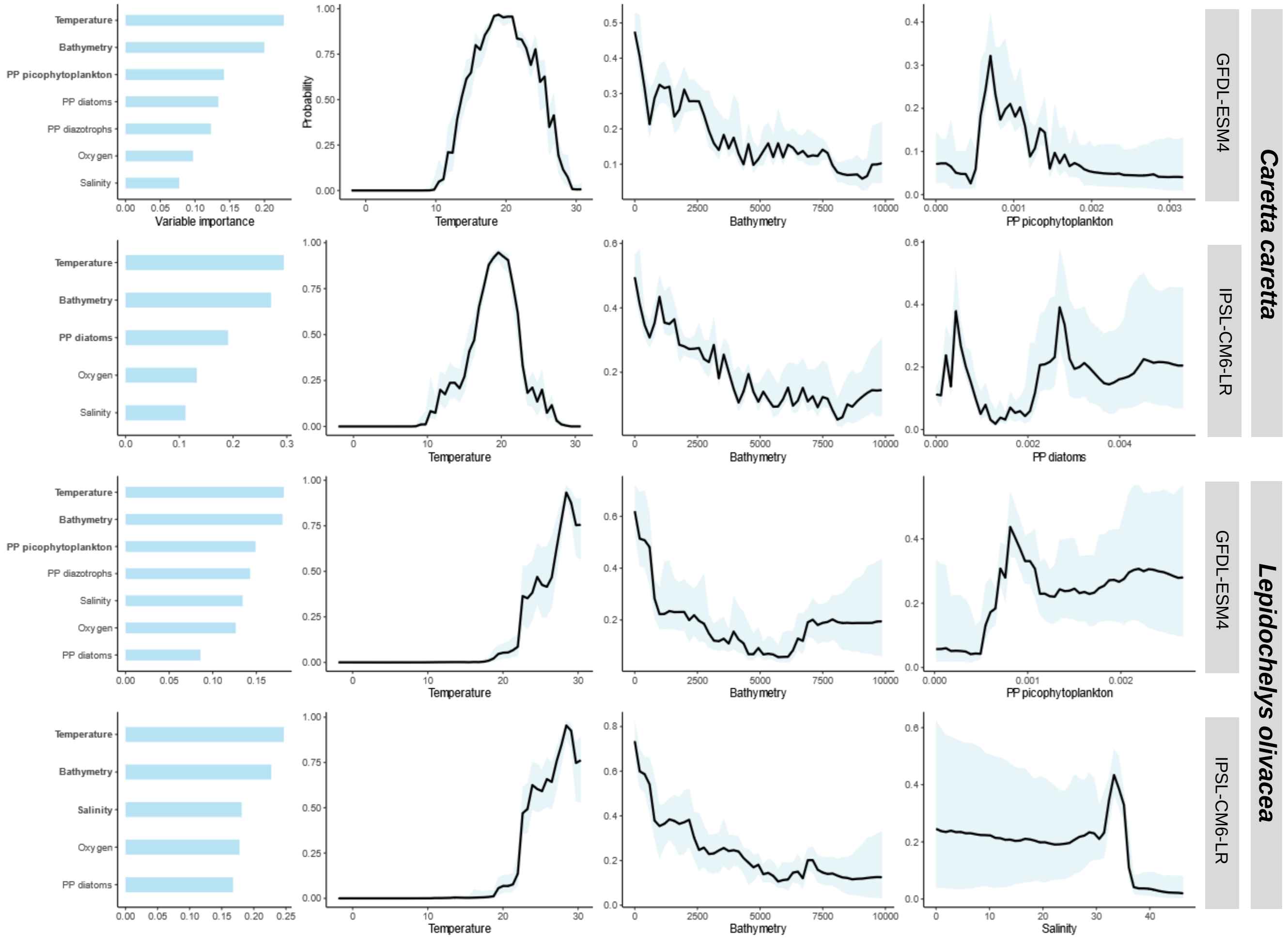

Figure S8. Results for the species *Caretta caretta* and *Lepidochelys olivacea*. The first column represents the contributions of all the variables to the model for both ESMs. We also provide the additive relation for the variables that have contributed the most to the model. These additive relations represent the probability of being present at some point along the x-axis.

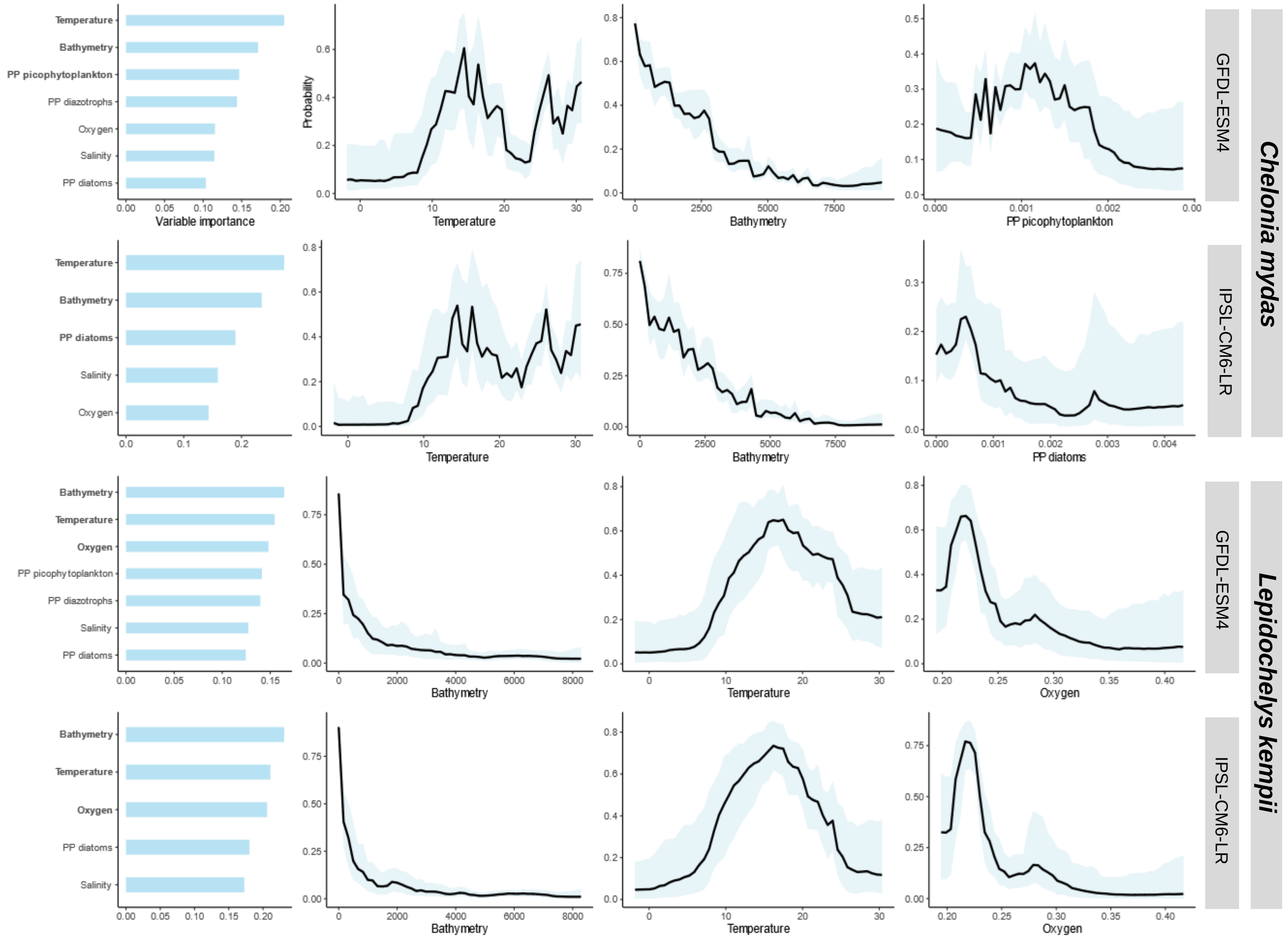

Figure S9. Results for the species *Chelonia mydas* and *Lepidochelys kempii*. The first column represents the contributions of all the variables to the model for both ESMs. We also provide the additive relation for the variables that have contributed the most to the model. These additive relations represent the probability of being present at some point along the x-axis.

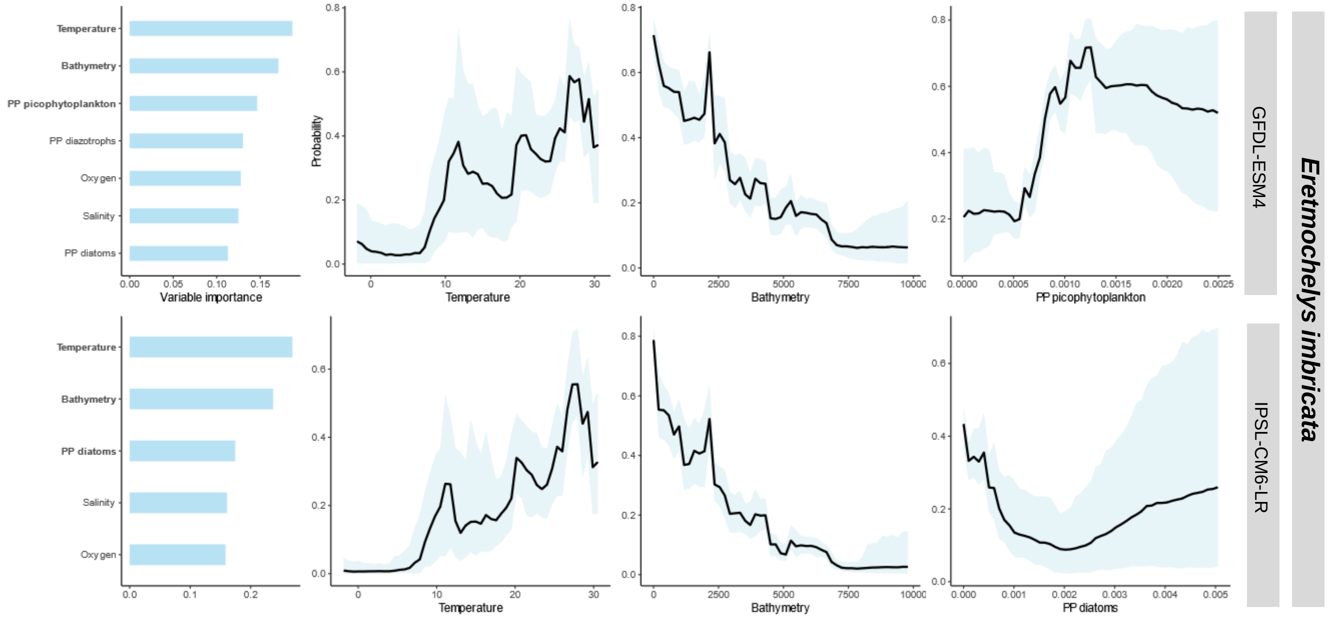

Figure S10. Results for the species *Eretmochelys imbricata*. The first column represents the contributions of all the variables to the model for both ESMs. We also provide the additive relation for the variables that have contributed the most to the model. These additive relations represent the probability of being present at some point along the x-axis.

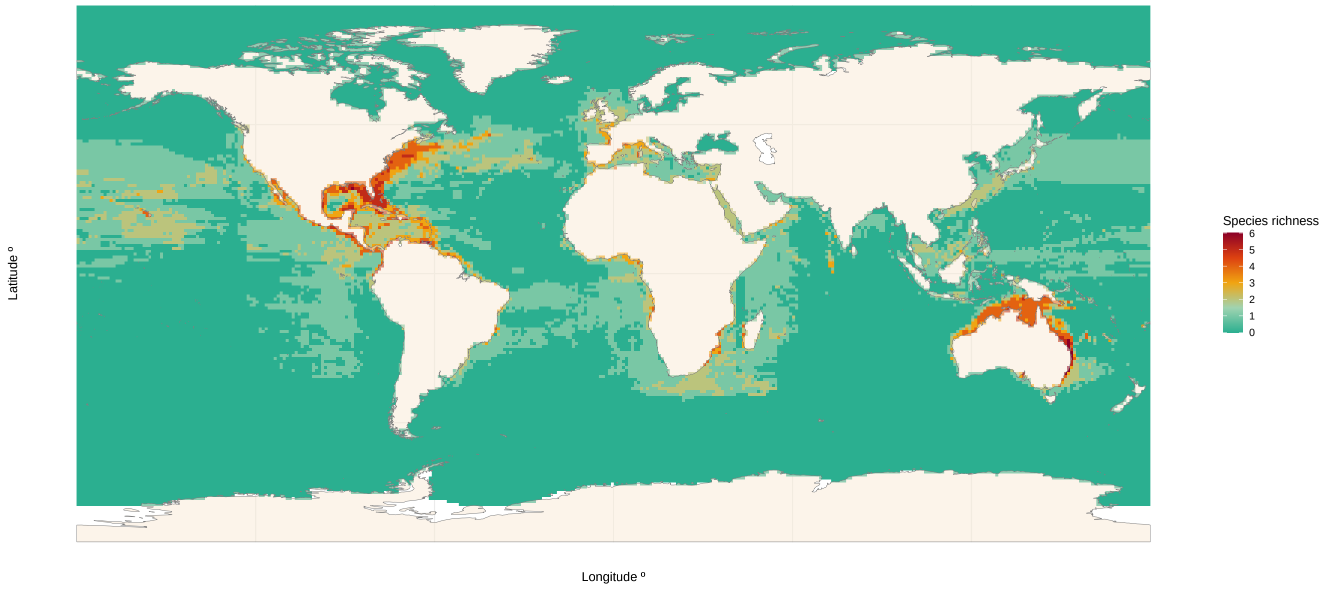

Figure S11. Global hotspots of marine turtle species richness for the historical native range (1950-2014). Warmer colors indicate higher species richness, highlighting areas with the presence of up to six species. This visualization emphasizes regions of ecological importance for conservation efforts.

3 Error measures

ESM	*N. depressus*	*D. coriacea*	*C. caretta*	*L. olivacea*	*C. mydas*	*L.kempii*	*E. imbricata*
GFDL-ESM4	0.95	0.70	0.84	0.85	0.84	0.95	0.89
IPSL-CM6-LR	0.94	0.65	0.78	0.81	0.81	0.93	0.86

Table S3. AUC measure for each species and ESM results (GFDL-ESM4 and IPSL-CM6A-LR).

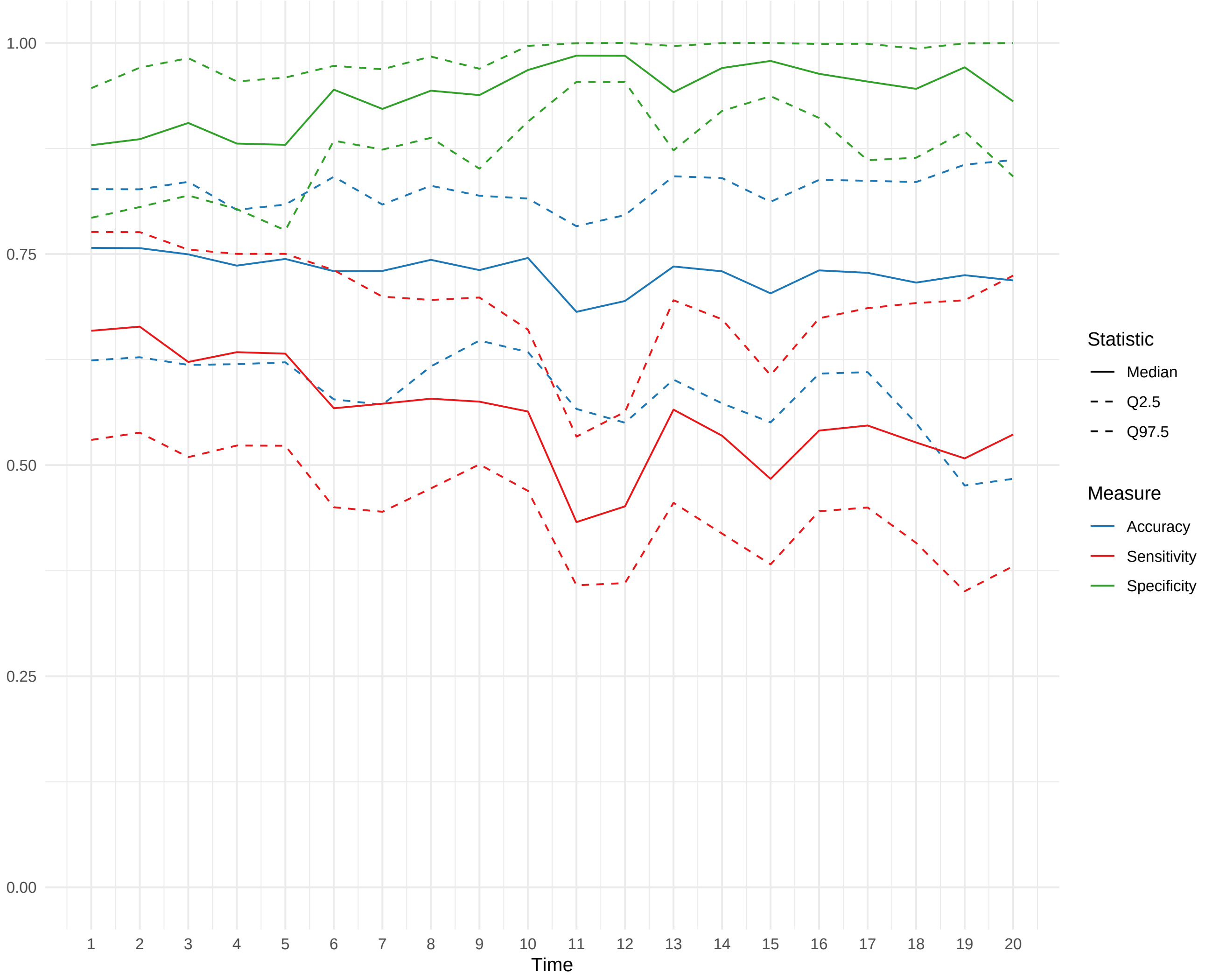

Figure S12. Results of error measure values for the performance of BART under a pseudo-absence and cosmopolitan species scenario. The red line represents sensitivity, the green line represents specificity, and the blue line represents accuracy. The dotted lines represent the 0.025 and 0.975 quantiles. These measures are calculated for each year of the simulation study.

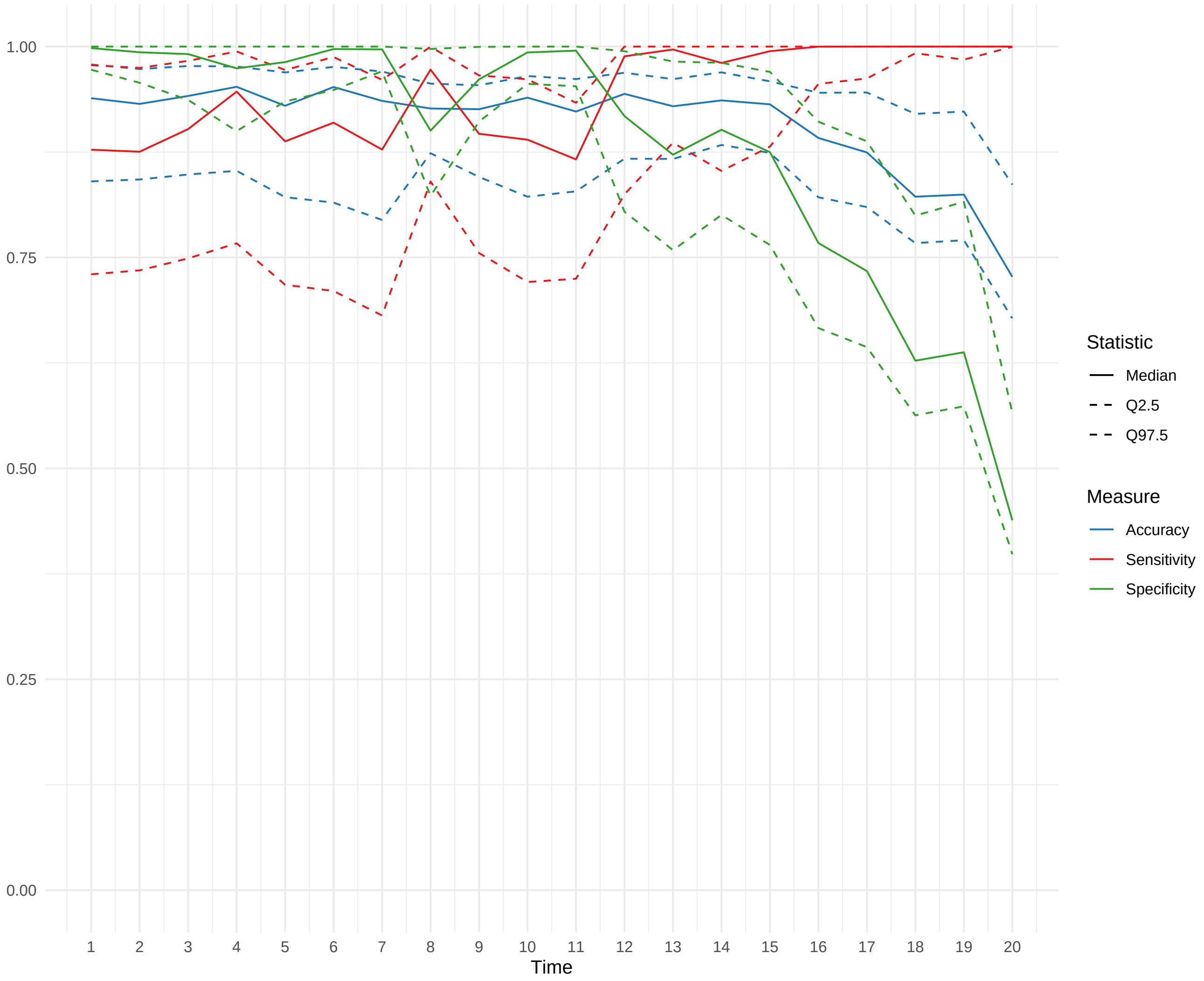

Figure S13. Results of error measure values for the performance of BART under a pseudo-absence and **persistent** species scenario. The red line represents sensitivity, the green line represents specificity, and the blue line represents accuracy. The dotted lines represent the 0.025 and 0.975 quantiles. These measures are calculated for each year of the simulation study.
